# Pulmonary inflammation in severe pneumonia is characterised by compartmentalised and mechanistically distinct sub-phenotypes

**DOI:** 10.1038/s41467-026-74190-x

**Published:** 2026-06-23

**Authors:** Mark Jeffrey, Josefin Bartholdson Scott, Shyamanova Mazumdar, Richard J. White, Ellen Higginson, Mailis Maes, Sally Forrest, Joana Pereira-Dias, Surendra Parmar, Emma Heasman-Hunt, Martin D. Curran, Petra Polgarova, Jurgen Herre, Els Wauters, Diether Lambrechts, Pierre Van Mol, Cato Jacobs, Joost Wauters, Emma E. Davenport, Stephen Baker, Gordon Dougan, Vilas Navapurkar, Andrew Conway Morris

**Affiliations:** 1 Division of Perioperative, Acute, Critical Care and Emergency Medicine, Department of Medicine, University of Cambridge, Cambridge, UK; 2 Cambridge Institute of Therapeutic Immunology & Infectious Disease, Department of Medicine, University of Cambridge, Cambridge, UK; 3 European Molecular Biology Laboratory, European Bioinformatics Institute (EMBL-EBI), Wellcome Genome Campus, Hinxton, UK; 4 Wellcome Sanger Institute, Wellcome Genome Campus, Hinxton, UK; 5 The School of Medicine, University of St Andrews, St Andrews, UK; 6 Clinical Microbiology and Public Health Laboratory, UK Health Security Agency, Cambridge, UK; 7 John V Farman Intensive Care Unit, Addenbrooke’s Hospital, Cambridge, UK; 8 Department of Respiratory Medicine, Addenbrooke’s Hospital, Cambridge, UK; 9 Laboratory of Respiratory Diseases and Thoracic Surgery (BREATHE), Department of Chronic Diseases and Metabolism, KU Leuven, Leuven, Belgium; 10 Department of Pneumology, University Hospitals Leuven, Leuven, Belgium; 11 Laboratory of Translational Genetics, Department of Human Genetics, KU Leuven, Leuven, Belgium; 12 VIB Center for Cancer Biology, VIB, Leuven, Belgium; 13 Laboratory for Clinical Infectious and Inflammatory Disorders, Department of Microbiology, Immunology and Transplantation, KU Leuven, Leuven, Belgium; 14 Medical Intensive Care Unit, University Hospitals Leuven, Leuven, Belgium; 15 A*STAR Infectious Diseases Labs (A*STAR IDL), Agency for Science, Technology and Research (A*STAR), Singapore, Singapore; 16 Centre for Translational Stem Cell Biology, LKS Faculty of Medicine, Hong Kong University, Hong Kong, China

**Keywords:** Infectious diseases, Translational research, Respiratory distress syndrome, Gene regulation in immune cells, Mucosal immunology

## Abstract

Pneumonia is the leading infectious disease killer worldwide and commonly requires admission to critical care. Despite its prevalence, the underpinning biology of severe pneumonia remains incompletely understood. Here we perform multifaceted assessments of bronchoalveolar transcriptome, cytokines, microbiology, and clinical features to biologically characterise a cohort of patients with suspected severe pneumonia. Our data implicate three lung-restricted transcriptionally defined severe pneumonia endotypes (termed ‘Pneumotypes’ (Pn)). All three Pneumotypes have comparable clinical presentations and severity of respiratory failure but experience divergent outcomes. Pn1, the most common, is characterised by low alveolar cytokines, expanded tolerogenic macrophages and epithelial damage. Pn3 is characterised by immature neutrophil infiltration, *IL-6-STAT3* activation and longer duration of mechanical ventilation. Pn2 displays the fastest resolution, exhibiting a balanced immune response and epithelial-endothelial repair signatures. We identify and validate mechanistically distinct phenotypes in the lungs of patients with suspected pneumonia and acute lung injury, implicating targets for personalised therapy.

## Abstract (web summary)

Analysis of cell samples from pneumonia patients may indicate specific phenotypes associated with disease. Here the authors use a multi-omics approach to analyse transcriptional pneumotypes in severe pneumonia showing different features and inflammatory profiles in these different groups including differences in neutrophil phenotypes and cytokine responses.

## Introduction

Pneumonia is the commonest infectious cause of death worldwide, responsible for an estimated 2.5 million deaths per year^1^ and second commonest cause of sepsis^2^. Severe pneumonia accounts for 60% of all infections managed in intensive care^3^. Pneumonia is also the most common trigger for acute respiratory distress syndrome (ARDS)^4^, which is associated with increased morbidity and mortality^5^.

Despite the considerable burden of pneumonia, the syndrome is incompletely understood and diagnosis is difficult. There is limited overlap between the clinical-radiological syndrome used for diagnosis and histopathologically confirmed pneumonia^6^. Distinguishing infection from sterile mimics remains challenging^7,8^. Blood-based biomarkers have poor diagnostic performance^9^, leading to the investigation of lung sampling to identify compartmentalised inflammation. Whilst alveolar cytokines, notably interleukin 1 beta (IL-1β) and CXCL-8, have demonstrated excellent sensitivity, they have poor specificity^8^ and failed to change antimicrobial prescribing in clinical trials^10^. Alveolar neutrophil counts are also sensitive but non-specific for pneumonia of bacterial origin^11^. Although the mechanisms driving alveolar inflammation remain unclear, the low specificity seen with cytokines and neutrophil counts imply common pathways terminating a diverse range of upstream insults.

Limited therapeutic advances in critical illness have led to attempts to move away from broad, clinically defined syndromes and towards pathophysiologically defined entities^12^. Conflicting results in trials of immunomodulatory therapies in pneumonia support this contention^13,14^. Peripheral blood phenotypes have been identified in sepsis arising from pneumonia^15^ and ARDS^16^. However, whilst these approaches predict outcomes and may explain some of the heterogeneity in therapeutic trials, to date, such an approach has not been applied directly at the site of infection, i.e. the lungs. This is despite the well-established compartmentalisation of inflammatory responses^8,17^.

In this work, we examine cohorts of ventilated patients with clinical pneumonia syndrome and profile the immune responses in bronchoalveolar lavage and blood compartments. We identify transcriptional and phenotypic heterogeneity and diverse disease processes that may be exploited to personalise future pneumonia therapy.

## Results

### Bronchoalveolar host gene transcription defines three sub-phenotypes in patients with suspected pneumonia

We recruited a cohort of 95 mechanically ventilated patients with clinically suspected pneumonia from a mixed medical-surgical intensive care unit (ICU)^18^. Eighty of these patients had sequenceable RNA from bronchoalveolar lavage cells. The onset of suspected pneumonia was a mixture of community and hospital-acquired. Overall, 34 (43%) had their pneumonia confirmed after expert consensus review, with bacteria being the most common aetiological agents (Table 1). In keeping with the syndrome of severe pneumonia, the patients had significantly impaired oxygenation, a high rate of acute respiratory distress syndrome (ARDS) and high severity of illness (Table 1). In-hospital mortality was 33%, which is typical of pneumonia requiring ICU admission and mechanical ventilation^19,20^. Immunosuppression rates were also high at 40%, although not dissimilar to previously published cohorts with severe pneumonia^21^.

**Table 1:** Clinical and demographic features of the overall cohort and individual Pneumotypes

Group		Overall *N* = 80	Pn1 *N* = 39	Pn2 *N* = 19	Pn3 *N* = 22	*p* value
Demographics	Age (Years)	60 (44, 70)	58 (42, 67)	56 (43, 73)	66 (57, 74)	0.061
	Female	34 (43%)	17 (44%)	8 (42%)	9 (41%)	>0.9
	BMI > 30 kg/m^2^	16 (20%)	6 (15%)	3 (16%)	7 (32%)	0.3
	Immunosuppressed	32 (40%)	22 (56%)	5 (26%)	5 (23%)	0.014
	Neutropenic	7 (8.8%)	4 (10%)	3 (16%)	0 (0%)	0.2
	Transplant recipient	6 (7.5%)	2 (5.1%)	2 (11%)	2 (9.1%)	0.6
	Admission APACHE II	16 (12, 23)	15 (12, 25)	16 (11, 22)	18 (14, 20)	0.8
	PF ratio	23 (16, 31)	26 (16, 35)	23 (15, 33)	20 (16, 24)	0.2
	ARDS	45 (58%)	22 (58%)	11 (58%)	12 (57%)	>0.9
	Shock	16 (20%)	10 (26%)	1 (5.3%)	5 (23%)	0.2
	Illness onset prior to BAL (Days)	-1.5 (-4.5, -1.0)	-1.0 (-6.0, 0.0)	-2.0 (-3.0, -1.0)	-1.5 (-6.0, -1.0)	0.8
	Antibiotic free days (Day 28)	10 (2, 20)	7 (3, 16)	21 (9, 23)	9 (0, 17)	0.013
	In-Hospital Mortality	26 (33%)	12 (31%)	5 (26%)	9 (41%)	0.6
	Ventilator free days (Day 28)	6 (0, 21)	8 (0, 21)	13 (0, 25)	0 (0, 10)	0.084
Blood results	White cell count (x10^9^/L)	11 (6, 19)	10 (6, 22)	10 (4, 13)	11 (10, 21)	0.15
	Neutrophil count(x10^9^/L)	9 (5, 16)	9 (4, 17)	8 (3, 9)	10 (8, 20)	0.048
	Lactate (mmol/L)	1.30 (1.00, 1.95)	1.60 (1.00, 2.20)	1.20 (0.80, 1.60)	1.35 (1.00, 1.80)	0.10
	CRP (mg/L)	187 (123, 280)	164 (55, 239)	169 (129, 246)	260 (145, 310)	0.073
Evidence	Radiological evidence of pneumonia	63 (79%)	33 (85%)	13 (68%)	17 (77%)	0.3
	Microbiological evidence of pneumonia	39 (49%)	17 (44%)	8 (42%)	14 (64%)	0.3
Aetiology	Cases adjudicated as pneumonia	32 (40%)	12 (31%)	6 (32%)	14 (64%)	0.029
	Bacteria pneumonia	20 (25%)	4 (10%)	3 (16%)	13 (59%)	<0.001
	Viral pneumonia	11 (14%)	8 (21%)	2 (11%)	1 (4.5%)	0.2
	Fungal pneumonia	3 (3.8%)	1 (2.6%)	1 (5.3%)	1 (4.5%)	>0.9
BAL quality	Library storage time (days)	329 (172, 454)	349 (170, 497)	363 (179, 454)	201 (83, 417)	0.2
Admission diagnosis	Admission diagnosis				0.12	
	Non-infectious non-respiratory organ failure	17 (21%)	5 (13%)	7 (37%)	5 (23%)	
	Non-infectious Respiratory Failure	17 (21%)	9 (23%)	1 (5.3%)	7 (32%)	
	Non-pulmonary Infection	8 (10%)	6 (15%)	0 (0%)	2 (9.1%)	
	Pulmonary Infection	35 (44%)	17 (44%)	10 (53%)	8 (36%)	
	Transplant	3 (3.8%)	2 (5.1%)	1 (5.3%)	0 (0%)	

Continuous values shown as median and interquartile range, categorical by n (%). BMI -body mass index, PF ratio- PaO_2_/FiO_2_ ratio, CRP -C-reactive protein. *P* value by Chi^2^ for categorical data or two-sided Fisher’s exact when cell values were <5. *P* values by two-sided Kruskal-Wallis one way analysis of variance continuous variables.

**Fig. 1:**
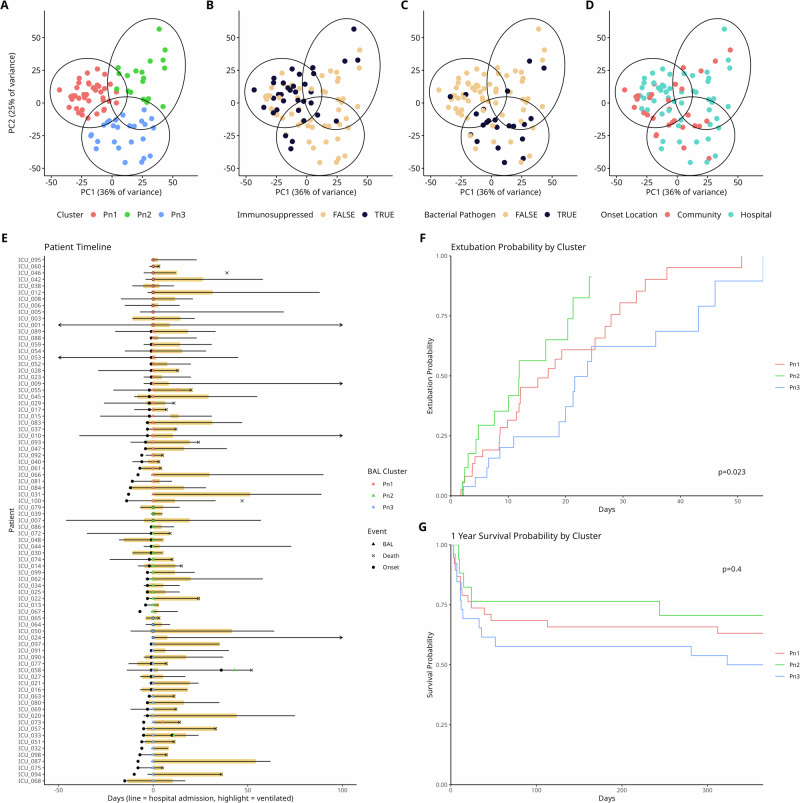
Bronchoalveolar Lavage transcriptomic clusters identifies 3 “Pneumotypes” with differing clinical characteristics. **A-D**Principal components 1 and 2 of the 10% most highly variable genes from bronchoalveolar lavage plotted with individuals coloured by: **A** Pneumotype identified through hybrid hierarchical k-means clustering from BAL bulk RNA transcriptome, **B** immunosuppressed state, **C** adjudicated bacterial pneumonia and **D** onset location. **E** participant timelines sorted by Pneumotype and time from illness onset to lavage. Triangle, coloured by Pneumotype, indicates lavage (red Pn1, green Pn2, blue Pn3), participants who were sampled twice have two lavage indicators. Solid line shows hospital admission. Yellow highlight indicates the period of mechanical ventilation, x indicates death. **F** Kaplan-Meier curves for time to extubation, censoring for death prior to extubation and **G** Kaplan-Meier Curves for survival to 1 year, p-value by log-rank test. *N* = 39 Pn1, 19 Pn2 and 22 Pn3. Source data are provided as a Source Data file.

To identify distinct pulmonary sub-phenotypes, we clustered patients based on their alveolar gene expression. Following sequencing of RNA from bronchoalveolar lavage and variance-stabilisation transformation^22^ the 10% most highly variable genes were clustered using the agglomerative hybrid hierarchical k-means algorithm^23^. This identified three clusters of patients with distinct pulmonary endotypes (Fig. 1A, Supplementary Fig. 1A–C), termed Pneumotypes 1, 2 and 3 (Pn1, 2, 3). Clustering did not occur due to technical factors such as batch or library size (Supplementary Fig. 2). Notably, all Pneumotypes displayed similar severity of respiratory failure, with the consistent proportion with ARDS (58%). Pn1 was enriched for immunosuppression (Fig. 1B, Table 1), whilst Pn3 was enriched for bacterial pneumonia (Fig. 1C, Table 1). Notably, neither of these features were exclusive to any Pneumotype, with Immunosuppression found in 26% and 23% of Pn2 and 3 respectively. Bacterial pneumonia was found in 10% and 16% of Pn1 and 2 respectively. Onset location in community or hospital were evenly distributed across the three (Fig. 1D).

Pneumotypes did not appear to reflect when the patient was sampled relative to disease onset (*p* = 0.8, Fig. 1E, Supplementary Fig. 3A), suggesting that these Pneumotypes were not different phases of disease evolution. In four cases patients were sampled twice, three during the same episode of pneumonia, demonstrating small positive migrations in principal component 2 (PC2) away from Pn3 (Supplementary Fig. 3B), whilst the fourth patient was sampled during two different ICU admissions with two distinct Pneumotypes.

Pn2 demonstrated significantly faster resolution of respiratory failure with shorter time to extubation when compared to Pn3 (HR for successful extubation relative to Pn2: Pn1 = 0.64 [95% CI: 0.32–1.25, *p* = 0.2], Pn3 = 0.31 [95% CI: 0.14–0.71, *p* = 0.006], Fig. 1F). Although the point estimate for 1-year mortality was lower in Pn2, controlling for age, the differences did not achieve statistical significance (HR for 1-year mortality relative to Pn2: Pn1 = 1.34 [95% CI: 0.52–3.47, *p* = 0.5], Pn3 = 1.44 [95% CI: 0.52–3.94, *p* = 0.5], Age 1.03 [95% CI: 1–1.05, *p* = 0.036], Fig. 1G).

**Fig. 2:**
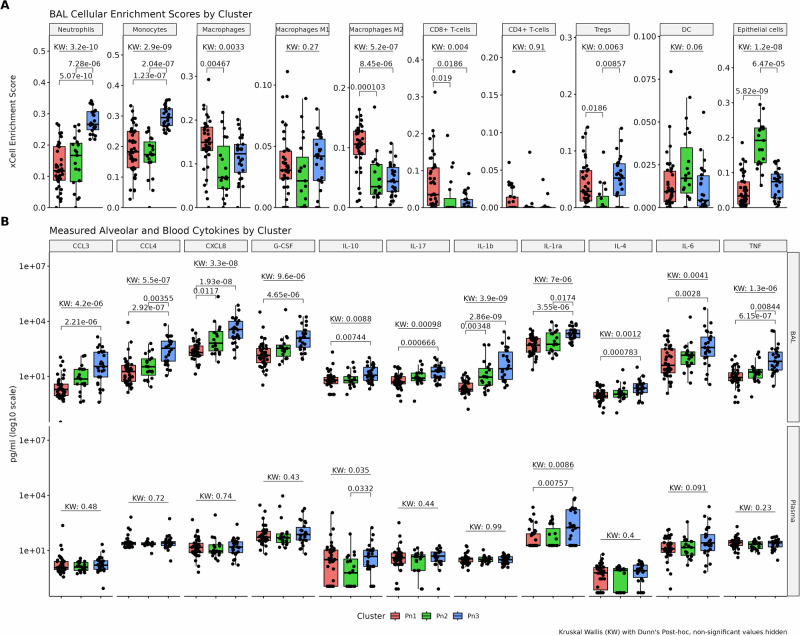
Estimated BAL cellular composition and comparison of BAL and plasma inflammatory proteins by Pneumotype. **A** Estimated proportional cellular composition from bulk RNA deconvolution using xCell by Pneumotype. Enrichment score is robust for comparisons within a cell type, but estimates transcriptional contribution rather than cell numbers so will be affected by relative RNA contents of cells when comparing between cell types. Identifies significantly elevated proportions of macrophages and monocytes in Pn3, M2 macrophages and CD8 + T-cells in Pn1 and elevated epithelial cells with reduced Treg proportions in Pn2. Box and whisker plots show median (central line), interquartile range (IQR) (box) and 1.5xIQR (whiskers) with individual data points shown as dots. *P* values by Kruskal-Wallis rank one way analysis of variance test (KW) and with Dunn’s post hoc pairwise test (non-significant pairwise values not shown for clarity). *N* = 39 Pn1, 19 Pn2 and 22 Pn3. **B** Comparison of selected BAL (top) and plasma (bottom) inflammatory protein concentrations by Pneumotype. Results for the full panel of measured proteins are shown in Supplementary Tables 1, 2. *P* values by Kruskal-Wallis rank one way analysis of variance test (KW) and with Dunn’s post hoc pairwise test (non-significant pairwise values not shown for clarity). Box and whisker plots show median (central line), IQR (box) and 1.5xIQR (whiskers) with individual data points shown as dots. *N* = 39 Pn1, 19 Pn2 and 22 Pn3 for BAL cytokines and *N* = 38 Pn1, 19 Pn2 and 22 Pn3 for serum cytokines. Source data are provided as a Source Data file.

We examined the differential cellular make-up of each Pneumotype by xCell bulk RNA deconvolution^24^ (Fig. 2A) and conventional cytology (Supplementary Fig. 4). Pn1 was characterised by an expanded macrophage population, driven by an increased representation of macrophages labelled ‘M2’, alongside expanded regulatory CD4 + T-cells (T_reg_) and cytotoxic CD8 T-cells. Pn3 demonstrated increased infiltrating peripheral blood immune cells (neutrophils and monocytes) and T_reg_. Pn2 showed expanded epithelial and dendritic cells with an intermediate representation of macrophages and neutrophils. Conventional cytology identified a comparable pattern of neutrophils and macrophages across Pn1-3 (Supplementary Fig. 4), although epithelial cells and monocytes were seldom identified. Comparison of total counts and unique genes sequenced did not identify any systematic differences between the pneumotypes, suggesting cellular composition did not drive RNA content (Supplementary Fig. 2).

We measured the concentrations of 48 inflammatory proteins, with 32 showing significantly elevated concentrations in the lavage of Pn3, relative to Pn1(Fig. 2B, Supplementary Table 1). Pn2 had intermediate inflammatory protein levels except for CXCL1(GROα) which was highest in Pn2. Notably, the concentrations of plasma inflammatory proteins were comparable across all three Pneumotypes, with only IL-1ra demonstrating a significant difference (Supplementary Table 2). Plasma and BAL protein concentrations were mostly weakly or very weakly autocorrelated (Source data file, Sheet 2). The strongest autocorrelation was observed for IL-6 (r = 0.59, Padj = 1e–7), whilst MCP-3 (r = 0.52), LTα and G-CSF (r = 0.51), IP-10 (r = 0.50) and CXCL1 (r = 0.45) also showed moderate autocorrelation.

**Fig. 3:**
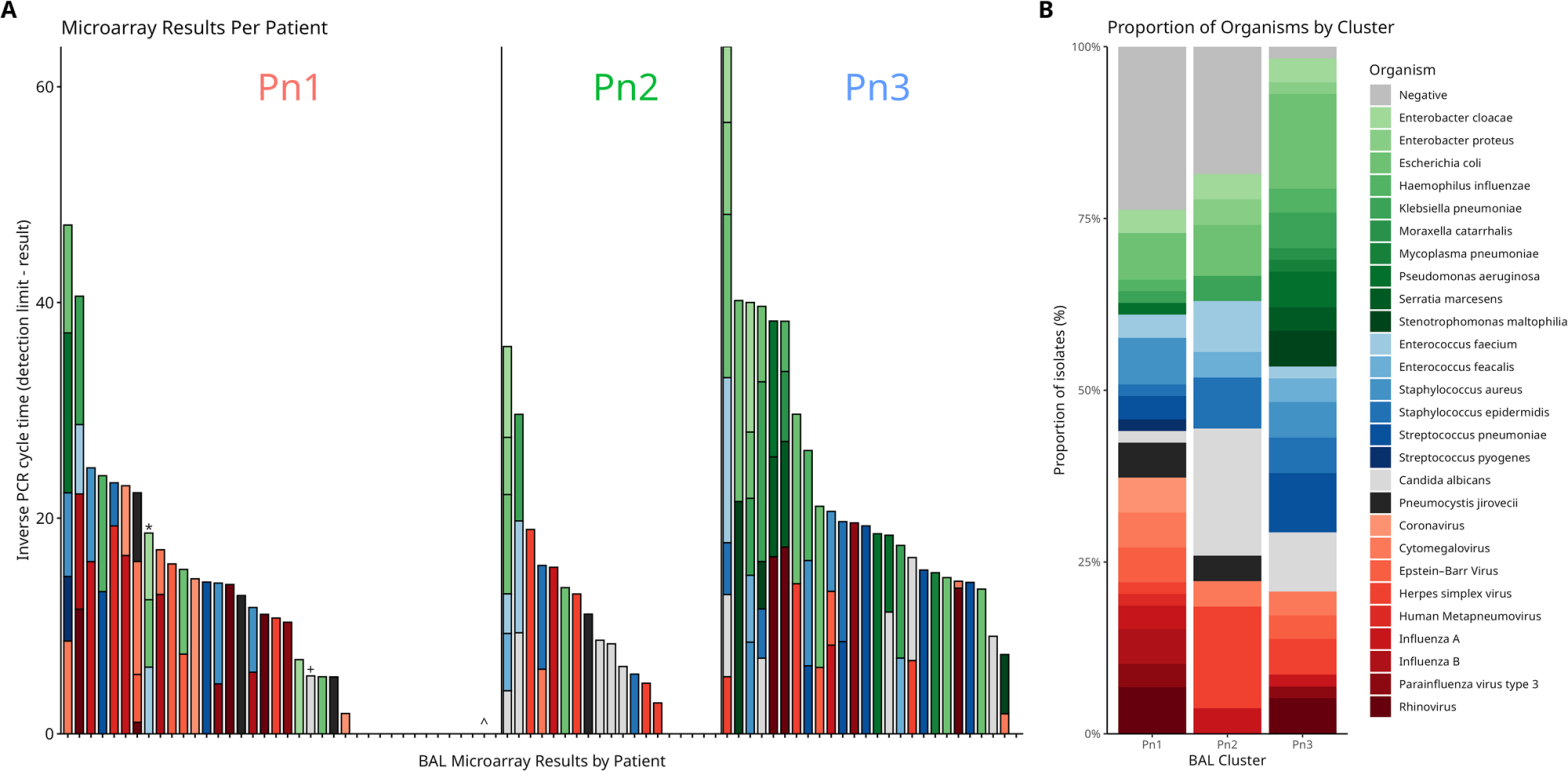
Pathogen PCR results by Pneumotype. **A** Pathogen TaqMan array card (TAC ‘microarray’) results per patient, symbols denote discrepancies between TAC, culture and sequencing: *denotes Citrobacter on sequencing but not culture or TAC, +denotes Rhinovirus on clinical PCR test but not TAC, ^ denotes Staph. Epidermidis >10^4^ CFU on culture and sequencing with negative TAC. **B** Pathogen TaqMan array card (TAC) detections summarised by Pneumotype and coloured by family (grey = negative TAC, greens = gram negative bacteria, blues = gram positive bacteria, black/white = fungi, reds = viruses). *N* = 39 Pn1, 19 Pn2 and 22 Pn3. Source data are provided as a Source Data file.

Respiratory pathogen associated nucleic acids were assayed on a 52-organism TaqMan Array Card^18^ (Fig. 3). These data were comparable with pathogen data extracted from metagenomic sequencing^18^. Pn3 had the highest proportion of pathogenic and non-pathogenic bacteria, with Pn1 being comprised more commonly of viral infections and samples with no pathogens detected. Pn2 had an increased proportion of low pathogenicity organisms (*Candida* spp., *Enterococci* and coagulase negative *Staphylococci*); however, once again no single organism type was exclusive to a given Pneumotype, and all Pneumotypes could be found in patients without an identified respiratory pathogen.

### Identifying mechanistic drivers of pneumotypes

#### Pneumotype 3 is characterised by inflammasome activation, expansion of immature neutrophils and impaired alveolar fluid clearance

**Fig. 4:**
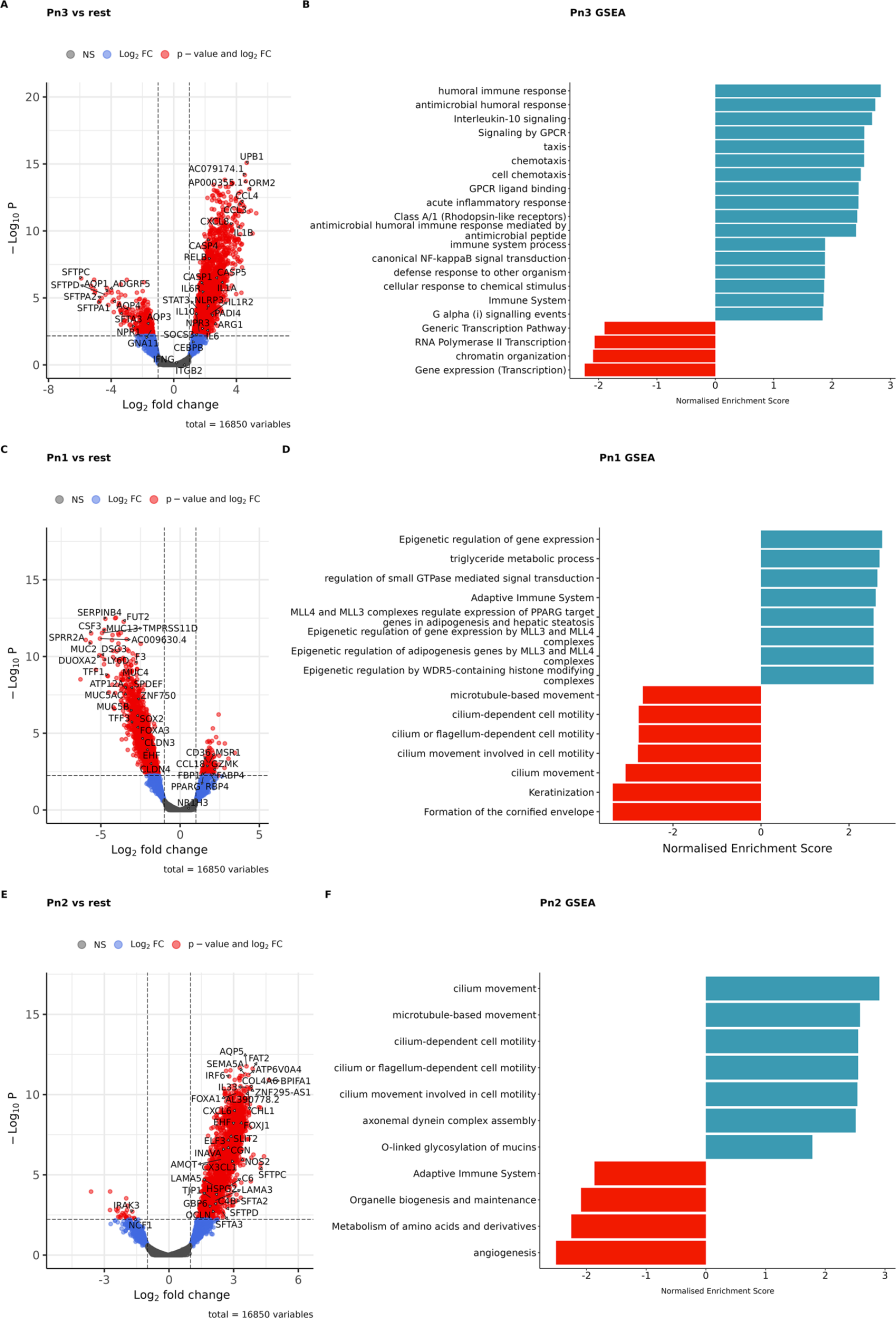
Transcriptional comparison of Pneumotypes. **A**,** B** Pneumotype 3. A Differential expression volcano plot for Pn3 vs rest. **B** Gene Set Enrichment Analysis (GSEA) of Gene Ontology Biological Pathways and ReactomePA terms showing the most up and downregulated pathways (dense rank ≤ 5) of differentially expressed genes for Pn3 vs rest **C**,** D** show these results for Pneumotype 1 and **E**,** F** for Pneumotype 2. Volcano plots indicate genes with Benjamini-Hochberg adjusted *p* value < 0.05 in red. *N* = 39 Pn1, 19 Pn2 and 22 Pn3. Source data are provided as a Source Data file.

To understand the mechanisms that underpin the Pneumotypes we examined differentially expressed genes (DEGs) between Pneumotypes in a 1-vs-all manner. Following adjustment for age, sex, library storage time and lavage return volume, 2,411 genes were differentially expressed in Pn3 vs Pn1 and Pn2 (Fig. 4A). Gene set enrichment analysis (GSEA) identified innate immune responses, neutrophil chemotaxis and type II interferon responses as among the most highly enriched pathways (Fig. 4B). Transcription factor (TF) enrichment for up- and down-regulated gene expression using Chea3^25^ (Supplementary Fig. 5A-B) identifies a network of pro-inflammatory transcription factors, including RELB and NFKB2, with enrichment of downstream genes including *NLRP3*, Caspases (*CASP 1,4,5*), *IL-1β* and *IL-6* (Fig. 4A). This is consistent with inflammasome activation and the high concentrations of alveolar cytokines identified (Fig. 2A, Supplementary Table 1). Alveolar neutrophilia was found in both deconvolution and cytology (Fig. 2A, Supplementary Fig. 4), with the enrichment of genes *IL1R2*, *PADI4* and transcription factor *CEBPB* implying an expansion of immature neutrophils with reduced antimicrobial function, with IL1R2 positive cells driving the maladaptive blood Sepsis Response Signature 1 pattern^26^. Both IL-1β and CXCL8, which play important roles in recruiting neutrophils to the alveolar space, were elevated at protein and transcript and protein level (Figs. 2B and 4A). Key mediators of emergency granulopoiesis, IL-6 and G-CSF^27^ were enriched at transcript and protein level, with both correlating with plasma levels (Supplemental Results file, Sheet 2), providing a link between the lung and the bone marrow release of immature neutrophils. Monocytes are key for sustained neutrophil recruitment^28^ and monocytes are also transcriptionally enriched in Pn3 (Fig. 2A) as are the monokines CCL3 and CCL4 (Figs. 2B, 4A, Supplementary Table 1). Although Pn3 is characterised by immune activation and infiltration of peripheral blood leucocytes, there is also evidence of concurrent immunoparesis. The elevation of counter-regulatory cytokines IL-10 and IL-1RA (Fig. 2B, Supplementary Table 1), expanded T_reg_ (Fig. 2A) and enhanced expression of conventional T-cell inhibitor Arginase-1 (*ARG1*), neutrophil inhibitory C5a-receptors (*C5aR1* and *2*) and negative co-stimulatory molecule *CD274* (*PDL1*) (Fig. 4A) are all indicative of simultaneous activation of counter-regulatory pathways.

Inspection of the down-regulated transcripts in Pn3 (Fig. 4A) identified genes involved in fluid clearance and alveolar surfactant function. Aquaporins (*AQP 1*,*3* and *4*) were down-regulated alongside atrial natriuretic peptide receptor 1 (*NPR1*), all of which play important roles in fluid clearance following acute lung injury^29,30^. Also notable amongst the suppressed transcripts were the surfactant proteins (*SFTPC*, *B, D, A1, A2* and *SFTA3*) alongside upstream receptor *ADGRF5*^31^ and intermediate signaller *GNA11*^32^. Loss of surfactant proteins are an established feature of acute lung injury^33^.

#### Pneumotype 1 demonstrates macrophage polarization, epithelial cytopathy and T-cell mediated pathology

Differential expression for Pn1 identified 1868 DEGs which were disproportionately down-regulated (Fig. 4C). GSEA reveals activation of stress response pathways with p53 signal transduction and MAPK signalling alongside lipid metabolism. Down-regulated pathways were predominantly those involved in epithelial and ciliated cell activity (Fig. 4D). The Chea3 analysis of up-regulated transcription factors in Pn1 indicated several lipid metabolism pathways involved in alternatively activated macrophage function, with enrichment of PPARγ, NR1H3 and NR1H4^34,35^ (Supplementary Fig. 5C). Alongside these transcription factors were multiple genes associated with alternatively activated tissue resident macrophages including *CD36, MRC1, CCL18, FABP4, FBP1, MSR1* and *RBP4*^36^ (Fig. 4C). These features are all consistent with the xCell deconvolution demonstrating expansion of ‘M2 labelled’ macrophages (Fig. 2A). Transcription factors associated with down-regulated genes include FOXJ1 and ELF3 (Supplementary Fig. 5D), both of which are involved in epithelial repair and indicate suppression of these pathways^37,38^.

As cells are thought to respond to relative levels and changes in cytokine concentrations, rather than absolute levels^39,40^, we examined relative levels of pro and anti-inflammatory/alternative activation polarising cytokines, finding IL-10, 13 and 4 were elevated relative to CXCL8, IL-1β and TNF-α but not IL-6 (Supplementary Fig. 6A).

Pn1 demonstrated low levels of bronchoalveolar neutrophils (Fig. 2A, Supplementary Fig. 4), however peripheral blood neutrophil counts varied considerably and this Pneumotype could develop in settings of both neutropenia and neutrophilia (Supplementary Fig. 6B). Although the expansion in CD8 + T-cells was noted in deconvolution (Fig. 2A), neither the TF mapping nor DEGs identified a clear T-cell signature. However, Granzyme K was enriched in Pn1 (Fig. 4C).

Examining the specific genes down-regulated in Pn1 and their associated TFs points to potential mechanisms of epithelial injury and consequent loss of barrier and gas exchange functions. This epithelial barrier damage is evidenced by the increased proportion of red blood cells in the lavage of these patients (Supplementary Fig. 4). The suppression of mucus production and processing genes (*ATP12A, FUT2, MUC4, MUC5AC, MUC5B*) alongside mucus-related transcription factors (*SPDEF and FOXA3*) and mucus components (*TFF1* and *TFF3*) indicate a loss of this important barrier component^41^. The loss of tight junction components claudin 3 and 4 (*CLDN3, 4*) (Fig. 4C) also points to impaired barrier function. Tissue factor (F3), the absence of which results in alveolar haemorrhage^42^, was also suppressed alongside the epithelial repair transcription factors *ZNF750* and *EHF* (Fig. 4C).

#### Epithelial repair and endothelial barrier function with a balanced inflammatory response characterise Pneumotype 2

Pn2 was associated with the best outcomes including the fastest time to extubation, a signal towards possibly lower mortality and the least use of antimicrobials in the time following lavage (Fig. 1F–G, Table 1). Although deconvolution indicates an expansion in epithelial cells (Fig. 2A), and DEG analysis points to genes involved in cilial function (Fig. 4E,F, Supplementary Fig. 7), the number of epithelial cells detected by cytology was minimal(Supplementary Fig. 4). Differential expression and GSEA (Fig. 4E,F) indicate cell motility and cilial processes. TF mapping by Chea3 indicates enrichment of FOXA1, FOXJ1, EHF, and ELF3 (Supplementary Fig. 5E). These have established roles in respiratory epithelial repair^37,38^ as well as IRF-6, an epithelial restricted interferon response protein with both barrier integrity and immune response functions^43^. Manual review of DEGs also identified upregulation of transcription factors *SOX2* and *SOX9*, associated with cellular stemness and regeneration including amongst distal alveolar stem cells (DASC)^44–46^. The TFs associated with down-regulated genes include CEPBE and SPI1 (Supplementary Fig. 5F), which point to the presence of mature neutrophils in contrast to the immature neutrophils seen in Pn3.

Manual review of gene expression also identified up-regulated endothelial gene expression in this Pneumotype (Fig. 4E). *SLIT2* and *ROBO*, which play a key role in preserving endothelial barrier function in infectious pulmonary insults^47^ were up-regulated alongside tight junction proteins *TJP1, OCLN, CGN* and tight junction regulators *AMOT* and *JUP1* and basement membrane components *HSPG2*, *LAMA3* and *LAMA5*.

Despite better outcomes in Pn2, we identified intermediate levels of lavage cytokines and inflammatory proteins (Fig. 2B), with gene expression enriched for cytokine signalling and innate immune function (Fig. 4E, F). Pn2 perhaps reflects the most adaptive Pneumotype of the three identified, showing a balanced immune response and pro-resolution epithelial and endothelial responses.

### Compartmentalisation of lung responses

**Fig. 5:**
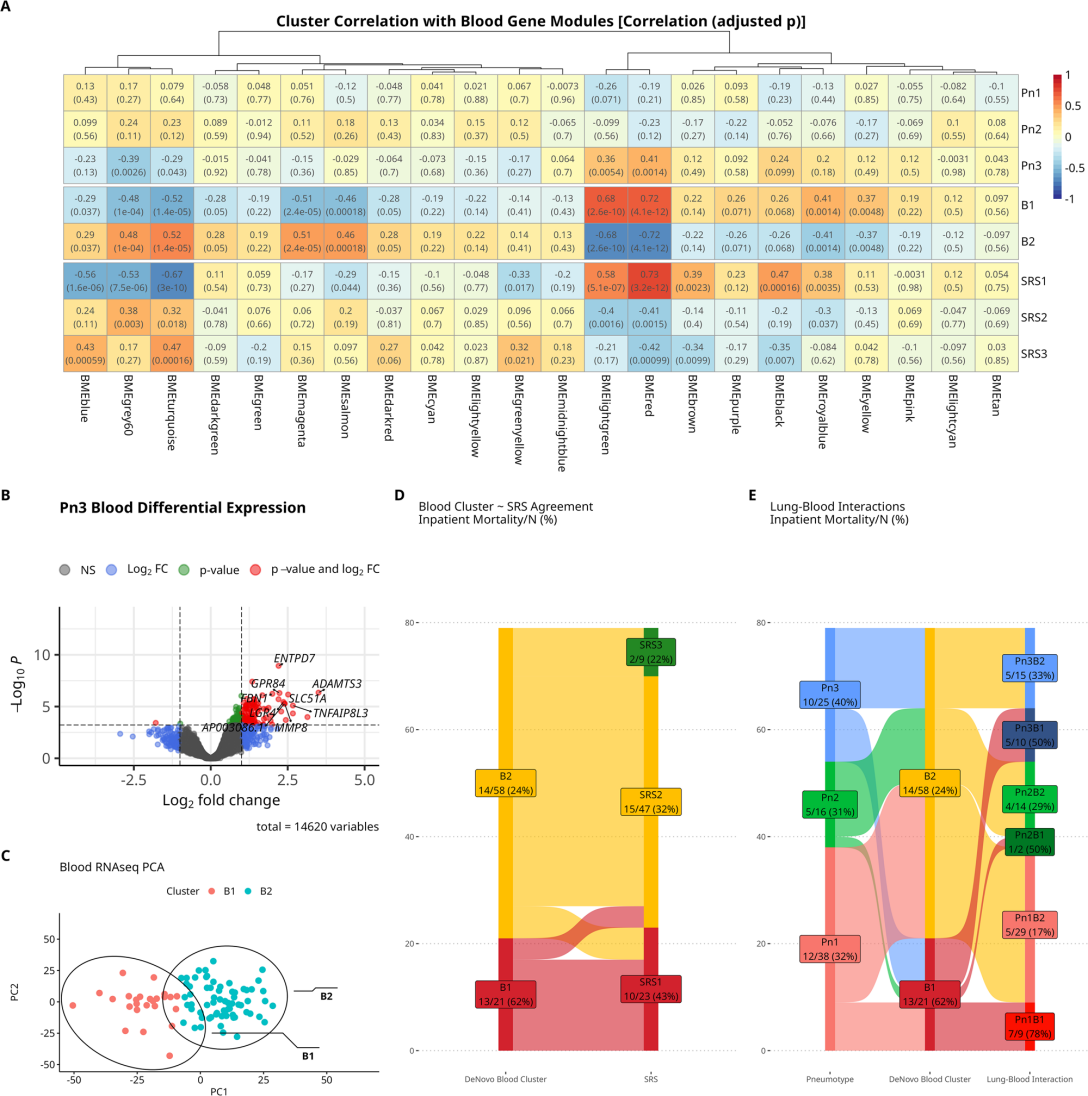
Transcriptional features of blood do not discriminate Pneumotypes, but do identify potential interactions between Blood and Lung transcriptomic subphenotypes. **A** Heatmap of lung and blood cluster correlations with blood gene co-expression modules. Upper 3 rows show Pneumotypes(Pn1-3), middle two Blood clusters (B1 and 2) and bottom SRS groups (SRS1-3)^15^. *N* = 74 participants with both blood and BAL RNAseq available. *p* value of correlations by Student’s asymptotic test, with Benjamini-Hochberg adjustment. **B** Differential expression volcano plot of Pn3 blood samples vs non-Pn3, *n* = 21. Volcano plots indicate genes with Benjamini-Hochberg adjusted *p* value < 0.05 in red (**C**). Principal components 1 and 2 of the highly variable blood genes coloured by blood cluster. **D** Alluvial plot of Blood and SRS assignments, percentage indicates inpatient mortality. **E** Alluvial plot of lung and blood cluster interactions and in-hospital mortality [n/total (%)]. *N* = 37 Pn1, 16 Pn2 and 21 Pn3. Source data are provided as a Source Data file.

We investigated gene expression in peripheral blood to determine whether Pneumotypes were associated with systemic responses, 74 of 80 patients with sequenceable BAL has sequenceable blood RNA. After filtering, 77% of expressed genes (13,540 common genes out of 17,625 total, 2586 unique to blood and 1499 unique to BAL) were identified in both blood and bronchoalveolar lavage. Blood RNA weighted gene correlation network analysis (WGCNA) (Fig. 5A upper three rows) demonstrated bland responses when segregated by Pneumotype. Further, no plasma inflammatory proteins differed significantly between Pneumotypes compared to 35/48 proteins measured in lavage (Fig. 2B, Supplementary Table 2). Differential gene expression in blood did not identify any significantly differentially expressed genes in either Pn1 or Pn2 when compared to not Pn1 or 2, and only 100 genes in Pn3 vs not Pn3 (Fig. 5B) which mapped to neutrophil de-granulation [*p* = 1.043e–15] and innate immune system [*p* = 6.346e–8] Reactome terms. Thus, identification of Pneumotypes from blood is unlikely to be feasible. For comparison we performed WGCNA of the BAL samples (Supplementary Fig. 7). This identified 9 modules with marked differences between the Pneumotypes with pathway mapping results consistent with the DEG analysis above (Fig. 4).

De-Novo clustering of the blood transcripts by HKMC identified two clusters (Fig. 5A middle two rows, 5 C, Supplementary Fig. 1D–F) with divergent outcomes and clinical features (Supplementary Table 3). The two clusters, termed Blood 1 (B1) and Blood 2 (B2), had distinct enrichment patterns with Blood 1 showing highly up-regulated coagulation (greenyellow module) and innate response, neutrophil de-granulation and TLR activation (pink) and moderately up-regulated interferon response (tan) modules. Down-regulated modules included RNA metabolic processes (turquoise), mitochondrial translation (lightgreen), nitrogen compound metabolic processes (blue) and erythrocyte homoeostasis (magenta) (Fig. 5A).

Our blood clusters were reminiscent of the previously described Sepsis Response Signatures (SRS)^15^. Examination of differentially expressed genes between Blood 1 and 2 to SRS 1 and 2 (SRS3 is assigned to healthy volunteers) identified a high degree of correlation (r = 0.83), reflected in similar patterns of WGCNA module correlation (Fig. 5A bottom 5 rows). Although our blood clusters and SRS assignments identified greater severity of illness in B1/SRS1, this was more pronounced in B1 and was reflected in a significant difference in mortality (Fig. 5D, Supplementary Table 3).

The distribution of blood phenotypes was uneven across the Pneumotypes, with Pn3 having proportionately more of the maladaptive B1 phenotype (in samples with valid blood and BAL RNAseq including re-sampled cases, B1 in Pn1 = 9/38 (24%), B1 in Pn2 = 2/16 (13%), B1 in Pn3 = 10/25 (40%), although 60% of Pn3 had a B2 blood phenotype.) Dyads formed by different Pneumotypes and blood phenotypes reveal divergent mortality outcomes (Fig. 5E), with the most notable difference seen in Pn1.

### Validation of pneumotypes

Although publicly available datasets that are directly comparable could not be identified, three cohorts of adult patients with suspected pneumonia with varying sampling, sequencing and clinical characteristics were identified with which to triangulate external validation. Two cohorts included mixed COVID-19 and other pneumonias; Wauters et al.^48^ investigated a cohort of 35 COVID-19 and pneumonia controls with single-cell RNA sequencing of low-volume (20 ml) BAL samples. Samples were analysed rapidly, without freezing, facilitating the sequencing of neutrophils, however severity varied compared to the discovery cohort and not all patients in this cohort were mechanically ventilated. Grant et al. collected BAL from patients with microbiologically confirmed pneumonia and undertook flow-cytometric cell identification alongside bulk RNA sequencing of sorted alveolar macrophages^49^. A third cohort reported by Langellier et al. collected tracheal aspirate (TA) from patients with suspected pneumonia and undertook bulk RNA transcriptomics^21^.

**Fig. 6:**
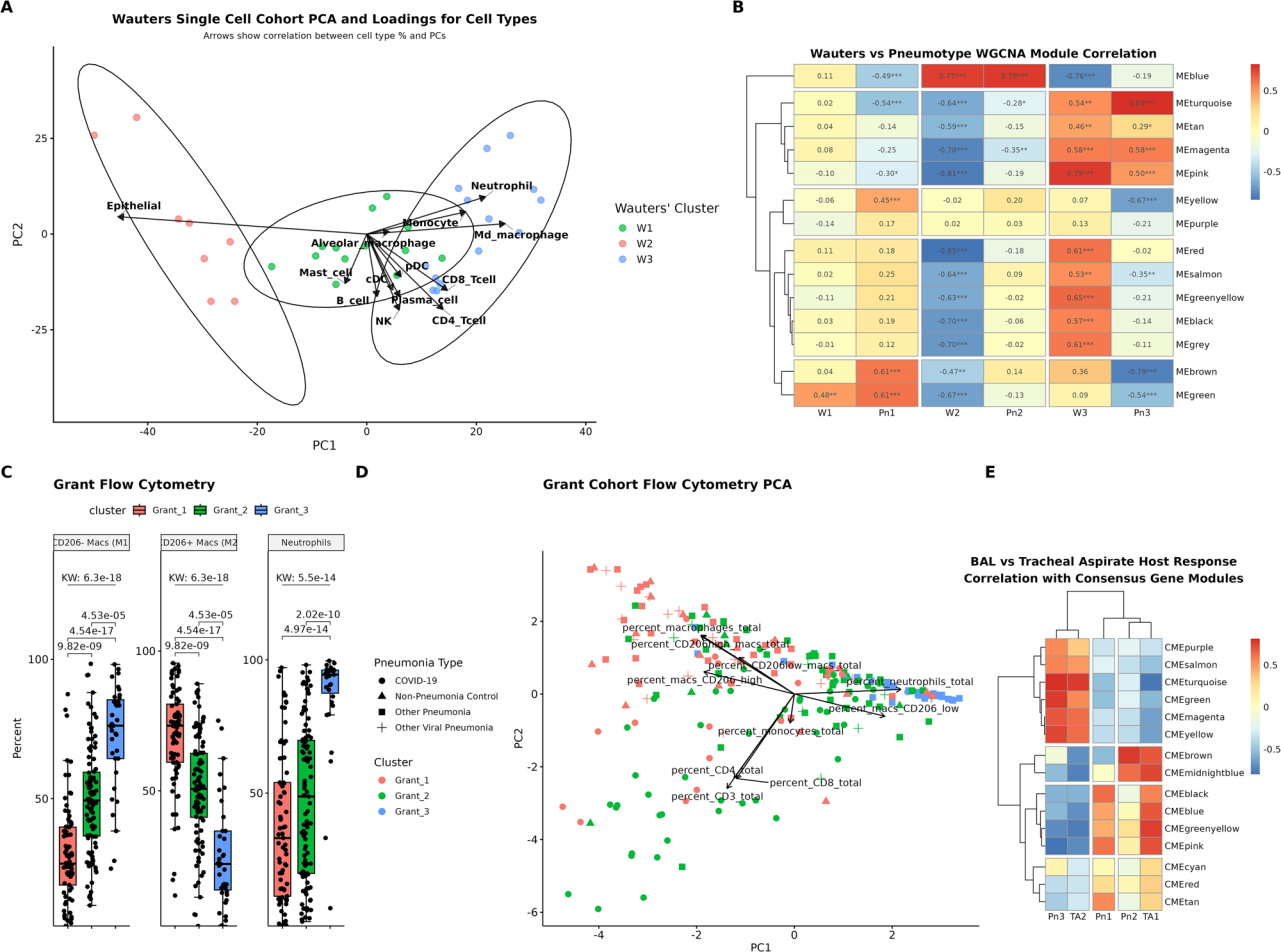
Validation of pneumotypes in external datasets. **A** Principal components 1 and 2 of the 10% most highly variable genes from bronchoalveolar lavage pseudobulked single-cell RNA sequencing from Wauters et al.^48^ plotted with individuals coloured by W-cluster (W1-3). Arrows indicate Pearson’s correlation between single-cell RNA annotated major cell type indicated and cluster. **B** Correlation of genes in W1-3 with the WGCNA modules identified in the discovery cohort (discovery cohort pneumotype correlations shown for comparison) with Student asymptotic *p* values. *N* = 13 W1, 8 W2, 14 W3 **C** Summary of flow cytometric data from Grant et al.^49^ with identification of CD206+ Macrophages, CD206- Macrophages, and neutrophils. *P* values by Kruskal-Wallis rank one way analysis of variance test (KW) and with Dunn’s post hoc pairwise test (non-significant pairwise values not shown for clarity). Box and whisker plots show median (central line), IQR (box) and 1.5xIQR (whiskers) with individual data points shown as dots. *N* = 59 for Grant 1, 78 for Grant 2 and 30 for Grant 3. **D** Principal Component Analysis of bulk macrophage RNA sequencing derived clusters from Grant et al. (Grant 1 red, Grant 2 Green, Grant 3 Blue) and diagnosis (circle COVID-19, triangle non-pneumonia control, square -bacterial pneumonia, cross non-COVID viral pneumonia). Arrows indicate Pearson’s correlation between percentage of flow cytometry annotated cell type indicated and cluster. **E** Tracheal aspirate from Langelier dataset^21^ and discovery cohort bronchoalveolar cell consensus gene co-expression modules derived from weighted gene co-expression network analysis (WGCNA) hierarchically clustered by similarity. *N* = 35 TA1 and 49 for TA2. Source data are provided as a Source Data file.

The single-cell data from Wauters^48^ was pseudobulked to simulate the sample type in the discovery cohort, with 11,216 genes passing filtering (10,748 (95.8%) overlapped the discovery cohort BAL genes, with 4289 (28.5%) from the discovery cohort not detected in the Wauters dataset). Using the 10% most variable genes, HKMC was conducted with K = 3, producing 3 clusters designated W1-3 (Fig. 6A). Cluster validity indices supported between 2 and 5 clusters in this dataset (Supplementary Fig. 1G–I). Gene expression in W2 correlated strongly with Pn2 (Pearson coeff r = 0.742), whilst W1 Pn1 and W3 Pn3 correlations were weaker (r = 0.135 and r = 0.384 respectively). Differentially expressed genes in these cluster pairings revealed moderate to high concordance in direction (W1/Pn1 149/154 shared DEGs, 96.8% concordance, W2/Pn2 2170/2177 shared DEGs, 99.7% concordance, W3/Pn3 2042/2410 shared DEGs 85% concordance). This data provided strong evidence for recapitulation of Pn2, with Pn1 and 3 less distinct. Correlation of normalised gene counts from the W clusters with the discovery cohort WGCNA modules revealed further similarities (Fig. 6B). W1 genes were positively correlated with Green module (lipid metabolic module, upregulated in Pn1), whilst W2 genes were positively correlated in Blue (cilial assembly genes upregulated in Pn2) and negatively with Turquoise and Magenta (innate immune modules, both down-regulated in Pn2). W3 genes were positively correlated with Turqouise, Magenta and Pink and negatively with Blue, although divergent signals were seen between W3 and Pn3 in Cyan, Brown and Salmon modules (Cell cycle, mitochondrial and translation modules respectively). The cellular composition of the W clusters was examined using the cellular annotations from the original single-cell sequencing (Fig. 6A PCA weightings and Supplementary Fig. 8A). This showed similar patterns to the Pneumotypes, with enrichment for alveolar macrophages in W1, epithelial cells in W2 and neutrophils, monocytes and inflammatory monocyte-derived macrophages in W3. Consistent with the correspondence with Pneumotypes, W1 was enriched for patients with immunocompromise, whilst W3 had more bacterial pathogens detected (Supplementary Table 4). To explore the hypothesised expansion of DASCs^44–46^ in Pn2 and IL1R2^+^ immature neutrophils in Pn3^26^, we examined the presence of these subsets in the Wauters dataset. Cells expressing DASC markers were found to be expanded in W2, whilst most immature neutrophil subsets, including the IL1R2^+^ subset, were expanded in W3 (Supplementary Fig. 8B)

By contrast to the Wauters data, the Grant dataset^49^ lacked the ability to detect Pn2 through lack of total bronchoalveolar cell bulk RNA sequencing and no epithelial cells identified by flow cytometry. Clustering the sorted bulk macrophage RNA sequencing identified three clusters designated Grant 1–3 (Fig. 6C, D, and Supplementary Fig. 1J–L), with flow cytometric counts identifying macrophage polarisation along the Grant 1 to 3 axis (PCA loadings in Fig. 6D) with inflammatory (CD206lo) macrophages, neutrophils and monocytes enriched in Grant 3, similar to Pn3. The paucity of neutrophils and increase in CD206hi alveolar macrophages in Grant 1 is similar to Pn1. 27 out of 36 (75%) of Grant cluster 3 had bacterial pneumonia, also consistent with Pn3 (Supplementary Table 5). The final cluster (Grant 2) in this data set was enriched for T-lymphocytes and has disproportionate numbers of patients with COVID-19. This is likely to be the COVID-specific phenotype Grant and colleagues identified in their original report^49^.

Examination of the endotracheal aspirate transcriptome reported by Langelier^21^ revealed an unsurprising paucity of alveolar cells, specifically lacking signals for macrophages and lymphocytes (Supplementary Fig. 8C). Clustering metrics indicate two clusters (Supplementary Fig. 1M–O). To compare the similarity of BAL and TA clusters, we generated a consensus gene co-expression network between Langelier’s and our cohort. These identified two tracheal clusters (termed TA1 and TA2) TA2 shared clinical and transcriptomic features with Pn3 positive correlation of Innate Immune WGCNA modules purple to yellow and negative correlation with metabolic modules black to pink (Fig. 6E), expanded neutrophils, monocytes and T_reg_ by xCell deconvolution and enrichment for bacterial pneumonia (Supplementary Fig. 8C and Supplementary Table 6). TA1 clustered with Pn2, with high correlation with cilial assembly module (brown) (Fig. 6E) and expanded epithelial cells (Supplementary Fig. 8A). However, some features of Pn1 were seen in TA2 (metabolic Black-Pink modules) with relative expansion of ‘M2 macrophages’ and restriction of neutrophil numbers. The inability to clearly distinguish Pn1 is likely due to paucity of macrophages in tracheal aspirate (Supplementary Fig. 8D).

## Discussion

Using bulk RNA sequencing of bronchoalveolar fluid we have identified three phenotypes in the lungs of patients with lung injury and suspected pneumonia. These phenotypes were reflected in the differential immune cell populations and inflammatory proteins. These phenotypes are compartmentalised to the lungs, are non-synonymous but interact with the peripheral blood immune phenotype and can be identified in external datasets drawn from pulmonary samples [21,48.49]. Each of these Pneumotypes is underpinned by distinct mechanisms and implies differential responses to therapies. They also bear comparison to recently described sub-phenotypes in the lungs of children with lung injury following bone-marrow transplantation^50^. Zinter and colleagues identified four sub-phenotypes, with differential alveolar cell types. One of these featured high levels of bacteria and neutrophils, like Pn3, although the remaining 3 sub-phenotypes did not match with those identified in our study. The differences in age and being bone marrow transplantation recipients may explain these divergent findings. Sarma and colleagues^51^ identified an 18-gene tracheal aspirate signature that distinguished clinical-laboratory defined hyper- and hypo- inflammatory ARDS from each other^16^. Neutrophils were dominant in both phenotypes. In contrast to our approach, Sarma and colleagues performed supervised analysis having dichotomised patients by peripheral blood cytokine and clinical features^16^. They also used tracheal aspirate, both of which may contribute to the findings of two rather than more pulmonary endotypes.

Each of the Pneumotypes contained both patients with and without confirmed pneumonia, implying common mechanisms underpinning lung injury arising from different mechanisms. This observation provides insight into previous failures to identify specific markers that distinguish pneumonia from other forms of lung injury^8^. The non-synonymous nature of the blood and bronchoalveolar phenotypes sounds a note of caution regarding the use of blood phenotypes alone to guide therapy^17^.

Pneumotype 3 is perhaps the most immediately recognisable Pneumotype, with its neutrophil dominant cytology, impairment of alveolar fluid clearance and loss of surfactant, it is closest to the classical description of pneumonia and ARDS pathophysiology^33^. In findings reminiscent of Kwok et al.’s description of the neutrophil phenotype in SRS1^26^, we found signals for enrichment of immature neutrophils in this setting and in the Wauters single-cell data set we identified potentially expanded IL1R2+ immature neutrophils in the W3 cluster. Immature neutrophils are known to have impaired antimicrobial functions but enhanced degranulation and consequent tissue toxicity^26,52^. The non-synonymous relationship between Pn3 and B1 (the latter being similar to SRS1) suggests that recruitment of immature neutrophils to the lungs in Pn3 may be selective and specific rather than simply reflecting peripheral blood left-shifted granulocytosis.

The neutrophil recruitment in Pn3, with IL-6 signalling sustaining emergency granulopoiesis and skewing of haematopoietic stem cells towards granulocyte production^27,53^, may form a positive feedback loop, creating a bi-stable equilibrium^54^ sustaining prolonged inflammation that can persist after the triggering insult is removed (schematic in Supplementary Fig. 9). The phenomenon of persisting inflammation after pathogen removal is well described, but poorly understood^55–57^. The presence of monocytes in Pn3 requires further confirmation, as although they were identified from the Bulk RNA signature, and in Grant and Wauters data, we did not confirm their presence by cytology. This may reflect the lower sensitivity of sampling a small number of cells by cytology, but does require further investigation. Overall, our findings suggest that patients with Pn3 may benefit from targeted immunomodulation alongside pathogen control. Approaches such as selective IL-6 blockade may be beneficial in Pn3, whilst conversely, may be harmful in Pn1 and of minimal benefit in Pn2.

Although Pn1 was characterised by macrophages enriched for an apparent pro-resolution transcriptional pattern, these patients have a similar degree and severity of lung injury. This observation illustrates the phenomenon of non-neutrophil induced lung injury. The existence of ARDS in neutropaenic patients has long been described^58,59^ but the mechanisms that underpin this syndrome have remained obscure^59^. In Pn1 viral pathogens, granzyme K release from CD8 + T-cells^60^ and further, as yet unidentified, factors may induce epithelial cytopathy. This leads to epithelial disruption and lung leak as exemplified by alveolar haemorrhage, differentiating this from neutrophil-driven damage in Pn3. Regarding the development and maintenance of Pn1, alternatively activated macrophages can exclude neutrophils from a tissue space^61^. Conversely, neutrophils themselves can induce a pro-inflammatory macrophage phenotype^62^. This phenotype may therefore arise from both an absence of peripheral blood neutrophils or polarisation of macrophages in the lungs (Supplementary Fig. 9). Pn1 therefore, appears to be maintained by tolerogenic macrophages, that are unable to clear pathogens, with consequent recruitment of CD8 cells that either alone, or in combination with macrophages and direct pathogen effects, induce cytopathic effects in alveolar epithelium.

Pn2 had prominent epithelial signatures in bulk RNA, although these were not clearly reflected in the cytology, raising the possibility of small numbers of highly transcriptionally active cells^63^. In exploring the Wauters data^48^, amongst the epithelial cells we identified distal alveolar stem cells (DASC) in W2. The ability of DASC to protect against lung injury in influenza models suggests these as a potential mediator of the protective phenotype in Pn2^46^.

In the 4 patients who were re-sampled, there was a consistent positive migration in Principal Component 2 (Supplementary Fig. 3B), away from Pn3 and towards either Pn1 or Pn2. However, we did not see an overall relationship between duration of illness and pneumotype (Supplementary Fig. 3A). Predictable transcriptional time related shifts in blood samples during sepsis occur^64^ and the observed migration from a neutrophil driven response to either recovery or immunosuppression fits with current models of immunopathology in sepsis^65^. Although this limited number of repeat samples should only be interpreted as hypothesis generating.

Although the phenotypes identified in the lungs are not well reflected in the blood, there are interactions that associate with different outcomes. Notably, the adverse blood phenotype is enriched in patients with Pn3, potentially explaining why previous studies appear to identify distinct lung phenotypes based on blood profiling in ARDS^16^. However, the work presented here demonstrates the need to assess both these compartments to understand the immunopathology and aid prognostication. Each compartment can be assigned a phenotypic category that combines to give an overall status (e.g. Pn1B1 or Pn2B1). Blood is a liminal fluid, connecting distinct tissue beds and allowing bi-directional interactions. Therefore, a fuller appreciation of the immunopathology in pneumonia, acute lung injury and indeed sepsis more widely is likely to require examination of other tissue compartments, most notably the bone marrow.

This study has several strengths, through its inclusion of a broad range of patients with diverse range of pathogens and sites of onset we can draw inferences about commonalities and differences between these groups. The phenotypes we have identified are robust to the clustering approach used and we can identify similar Pneumotypes in external datasets. There remain several areas of uncertainty. First, although we have constructed a triangulated validation in external datasets, the overlaps within each validation dataset are incomplete and further validation and refinement of these Pneumotypes in a full replication cohort using the same inclusion, sampling and analysis techniques is required. Factors such as the use of tracheal aspirate^21^, low volume lavage^48^ or sequencing isolated macrophages^49^ as well as clinical factors such as severity of illness^48^ and predominance of COVID-19 as a precipitant^48,49^ may have limited the ability to fully recapitulate our original findings. Whilst the temporal relationships between disease onset and sampling, and the few serial samples we have, do not point to the Pneumotypes being features of a common pathway sampled at different times, serial sampling will be required to confirm temporal stability and understand phenotype evolution and recovery trajectories.

Although we have identified three Pneumotypes, it is likely that other Pneumotypes may exist and may be identified in larger cohorts or those with distinct triggering pathologies, such as the potentially distinct T-cell driven responses in COVID-19 reported by Grant et al.^49^.

In conclusion we have identified and validated three pulmonary-confined endotypes in patients with severe pneumonia and lung injury. These phenotypes are underpinned by distinct mechanisms and have differential outcomes. The mechanisms point to different therapeutic options, as well as extending our understanding of the biology of lung inflammation in the context of severe pneumonia.

## Methods

### Design/setting/participants

#### Ethical approvals

The discovery cohort study was approved by the Leeds East Research Ethics Committee (17/YH/0286), Cambridge University Hospitals NHS Foundation Trust was the sponsor, and registered with clinicaltrials.gov (NCT03996330). The protocol has been deposited on Zenodo (doi/10.5281/zenodo.5081879). Written informed consent was obtained from participants or proxy assent, with retrospective consent sought from particpants who regained capacity whilst in hospital. Participants were not remunerated for participation in this study. Ethical approval for the publicly available datasets used, Wauters^48^, Langellier^49^ and Grant^21^ and colleagues, are set out in the original reporting manuscripts.

The discovery cohort study has been described previously^18^. Of the 95 patients recruited, sequenceable RNA from lavage cells that passed quality control was available from blood in 92 and BAL in 80 (5 patients were sampled twice with 4 having sequenceable RNA in the second sample), with both available for 74 patients. Recruitment was by consecutive participant availability and thus sex distribution reflected the patients in intensive care and is reported in Table 1. Sex was determined by assignment at birth and was included as a factor in differential gene expression.

Participants were recruited from a 20-bedded teaching hospital Intensive Care Unit (ICU). The unit is a mixed general medical-surgical unit which supports transplant and haematology-oncology services. Eligibility criteria were age ≥18, on mechanical ventilation, where the treating clinician suspected pneumonia and planned to undertake a diagnostic bronchoalveolar lavage (BAL). Exclusions were contraindications to bronchoscopy (e.g. by FiO2 > 80%, severe hypercapnia, coagulopathy or presence of small diameter endotracheal tube) or lack of informed consent or proxy assent.

Eligible patients were included consecutively when the study team was available (the study team were routinely unavailable from Friday 5 pm to Monday 8am, and also sporadically unavailable due to leave).

#### Sampling

Patients underwent bronchoscopy and lavage as per the unit’s standard operating procedure. Following wedging of the scope in a radiologically affected subsegment, up to 200 ml of saline were introduced in aliquots. The first aliquot of non-cellular material was discarded, and the remaining fluid processed for routine microbiology, Taqman array card (TAC), microbial sequencing, host cell RNA sequencing, cytology and inflammatory protein assays. Simultaneous draw of arterial or venous blood from indwelling lines was collected into Paxgene tubes for RNA preservation (Preanalytix, Hombrechtikon, Switzerland). A further blood sample was collected into EDTA (Sarstedt, Nuembrecht, Germany) and used for plasma generation with downstream inflammatory protein assays.

### Isolation of host BAL cells

BAL was centrifuged at 700 x *g* for 5 min to pellet cells, cells were then resuspended in saline, counted and 2.5 × 10^4^ cells were loaded in Shandon EZ Cytofunnels (ThermoFisher, Waltham, MA, USA) and spun onto cytoslides in a Cytospin cytocentrifuge at 32 × *g* for 4 min. The slides were fixed in methanol for 5 min and cells were stained using Shandon Kwik-Diff stain (ThermoFisher) and counted manually. The remaining cells were centrifuged again, and the pellet was resuspended in 350ul RLT buffer (Qiagen) with 1% beta-mercaptoethanol and stored at –70 ^o^C until RNA extraction.

### RNA extraction and sequencing

RNA from BAL cells was extracted using an RNAeasy kit (Catalogue number 74104 Qiagen, Venlo, Netherlands), and RNA from blood stored in PAXgene tubes was extracted using a PAXgene Blood RNA Kit (Catalogue number 762174 PreAnalytiX), following manufacturer’s recommendations. Sequencing libraries were constructed using an NEB Ultra II RNA custom kit (Catalogue number E7770L New England Biolabs,Ipswich, MA, USA), cDNA was amplified with dual indexed tag barcodes (14 cycles) (Eurofins, Luxembourg), then purified using Agencourt AMPure XP SPRI beads (Beckman Coulter, Brea, CA, USA). Libraries were pooled in equimolar amounts (20-plex), normalised to 2.8 nM and sequenced on the HiSeq 4000 platform (Ilumina, San Diego, CA, USA), to generate paired-end read lengths of 75 bp. Reads were mapped to the Genome Reference Consortium human build 38 (GRCh38) using Spliced Transcripts Alignment to a Reference (STAR) with read counts annotated using Ensembl 99. Following removal of the 12 haemoglobin genes from the blood samples, quality control checks were performed with FASTQc and Quality of RNA-Seq Toolset (QoRTs), resulting in the rejection of two lavage samples and two blood samples.

### Inflammatory protein analysis

Lavage supernatant and plasma inflammatory proteins were assayed using a Bio-Plex Pro Human Cytokine Screening 48-plex kit on a Bio-Plex 200 System (Catalogue number 12007283, Bio-Rad, Hercules, CA, USA), following manufacturer’s recommendations.

### Microbiological assays

The microbiological processing for conventional culture, TAC (ThermoFisher) and sequencing have been described in detail previously^18^. Briefly, samples were processed in accordance with the UK Standards for Microbiology Investigations (SMI) for conventional culture, alongside in-house PCR for respiratory viruses (adenovirus, enterovirus, human metapneumovirus, influenza A virus, influenza B virus, parainfluenza virus, rhinovirus, and respiratory syncytial virus), *Pneumocystis jirovecii* and herpesvirade (Herpes Simplex virus, Human Cytomegalovirus and Epstein Barr virus). The TaqMan array encompassed validated assays for 52 pathogens, with full details of coverage, development and validation with metagenomic sequencing reported previously^18^.

### Adjudication of pneumonia

Diagnosis of pneumonia was independently assessed by 2 experienced clinicians with access to clinical, radiological and microbiological data who used pre-agreed criteria to independently rate cases as ‘definite’, ‘highly likely’, ‘unlikely’ or ‘not pneumonia’. Any disagreement was resolved by a 3rd clinician. ‘Confirmed pneumonia’ was defined as consensus of ‘definite’ or ‘highly likely’ in keeping with previous studies^66,67^. Clinicians were blinded to host RNA and metagenomic sequencing results. The diagnostic components were summarized by assessing clinical, radiological, and microbiological criteria and the presence of systemic inflammation as defined by >=2 SIRS criteria (WCC < 4 or >12, temp <36 or >38 degrees C, HR >90bpm, RR >20bpm). Clinical criteria were defined by an increase in frequency or volume of respiratory secretions, increased oxygen requirement, deterioration in compliance, or signs of pneumonia on clinical examination. Radiological criteria where new or worsening pulmonary infiltrates or consolidation on X-ray or Computed Tomography (CT) imaging not explained by another cause. Microbiological criteria were positive blood, sputum, or BAL culture for known respiratory pathogens, serological or urinary pneumococcal or legionella antigen, TAC detection with cycle time (CT) ≤ 32^18^. The patient’s location 48 hours prior to the onset of the illness being investigated was recorded as community or hospital.

### Clinical parameters

Baseline demographic information including age, sex, body mass index, comorbidities and primary reason for ICU admission was recorded. Admission APACHE 2 score and PaO2/FiO2 ratio, white cell count (WCC) and C-reactive protein (CRP) immediately prior to bronchoscopy were recorded. Immunosuppression definition was based on the recent consensus statement^68^ and consisted of neutropenia, haematological malignancy, HIV infection with detectable viral load/CD4 count <250, current administration of immunosuppressive medications including corticosteroids >20 mg prednisolone equivalent and solid organ or bone marrow transplant. Patient outcomes were determined by electronic patient record (EPR) review after sufficient time for NHS spine updates to determine mortality up to 1 year and included duration of hospital admission and survival to nearest day, mechanical ventilation (end of last recorded period of mechanical ventilation) to nearest hour. Hazard ratios were calculated using Cox regression, with survival adjusted for age.

### Data management

Clinical data was collected from the EPR and recorded in a secure database. Patients were assigned unique study identifiers and identifiable information removed prior to analysis. Anonymized data and analysis code are made available with this publication at https://gitfront.io/r/mark-jeffrey/eZYpfcdwm7Eu/vapR/.

### Potential sources of bias

Sources of bias with limited recourse for control were patient fitness for bronchoscopy and single-centre recruitment. Although bronchoscopy forms part of the routine diagnostic workup for severe pneumonia in the trial unit, patients with difficult ventilation, on >80% oxygen or with significant coagulopathy will have been excluded by the treating clinicians.

To minimize batch effects during RNAseq, prepared libraries were stored frozen and sequenced as one batch. As this meant prolonged storage time for early samples, this was recorded. In addition, bronchoalveolar lavage has variable concentrations of RNA compared to blood. To assess the impact of this, return volume and whether lavage volume was <200 ml was also recorded. Impact of technical and clinical co-variates was assessed by variance partitioning and principal component analysis, with final differential expression model controlling for age, sex, freezer time and return volume.

### Bioinformatics and statistics

#### RNAseq quality control

Batch effects and outliers checked for using Hierarchical clustering and scatterplots of non-zero genes by library size. Filtering was performed using the edgeR function filterByExpr which keeps genes with a Counts Per Million (CPM) >= minimum count divided by median library size multiplied by 1e6. After assessing CPM density plots pre and post-normalization, a filtering threshold minimum count of 20 in at least 10% of samples was set for BAL. Blood samples were less sparse and the default minimum count of 10 was appropriate. Variance stabilizing transformation^22^ was applied prior to further analysis, with the exception of xCell deconvolution where TPM normalization was used on the advice of the package authors^24^. In total 60,664 unique genes were sequenced, with 15,039 post-filtering in BAL and 14,620 in blood.

#### Clustering

The 10% most variable genes were used for clustering. Agglomerative, hybrid hierarchical k-means (HKMC) clustering using Euclidean distance and Ward’s method with 10 iterations of k-means consolidation was performed^23^. Three clusters in BAL and two in blood were identified based on elbow plots, a local maximum in the gap statistic (for BAL) and silhouette score (Supplementary Fig. 1A–F). The cluster metrics for Wauters^48^ indicated between 1 and 5 clusters (Supplementary Fig. 1G–I), and 3 clusters were selected to recapitulate our initial analysis for validation. The Grant sorted macrophage cohort^49^ clearly identifies 3 clusters on elbow plot and gap statistic, though the maximum silhouette score was at 2 (Supplementary Fig. 1J–L). In the Langelier Tracheal Aspirate cohort^21^ the elbow plot and silhouette score identify 2 clusters, with a continually increasing gap score suggesting these may not be well separated (Supplementary Fig. 1M–O). HKMC was chosen over hierarchical and k-means clustering due to greater stability assessed by average pairwise Rand index on 100 bootstrapped samples with replacement^69^. Downstream clinical, microbiological and inflammatory protein features were robust to clustering method.

#### Deconvolution

Bulk RNA deconvolution to estimate cellularity was performed using xCell^24^. xCell performs cell type enrichment analysis for 64 cell signatures, pretrained on high-quality data. As recommended by package authors, TPM normalized gene counts were used for enrichment analysis, and spillover compensation utilized the default alpha=0.5. For comparison of BAL and TA samples, common genes raw counts were merged prior to TPM normalization—though without sample overlap this analysis should be interpreted with caution as batch effects cannot be assessed.

#### Differential expression

Differentially expressed (DE) genes between clusters were identified using an edgeR^70^ and Limma^71^ workflow. edgeR uses a negative binomial distribution, and robust, quasi-likelihood dispersions were estimated after effective library size calculation.

Model design was informed by variance partitioning, principal component analysis and WGCNA module correlation with technical variables, the final model was 0 + cluster + age + sex + library storage time + BAL return volume. Library storage time was significantly correlated with WGCNA modules related to cell cycle, whilst BAL return volume explained 14% of variance in PC1, which was co-correlated with predicted macrophage proportion.

DE was tested relative to a log2 fold change threshold >1. The resulting p-value histogram for one vs rest was bimodal. This could not be rescued by more stringent gene filtering and is likely a product of DE testing on clusters, as these are defined by the variance of the dataset and inherently paired and complementary with respect to gene expression, reassuringly global significance testing using a quasi-likelihood test produced the desired anti-conservative pattern. Pi0 will therefore be inflated for one vs rest comparisons, resulting in overly conservative false discovery rate (FDR) correction and increased risk of type 2 error. FDR *p* value threshold was set to 0.05.

#### Weighted gene co-expression network analysis (WGCNA)

WGCNA^72^ analysis was performed on the BAL, blood and validation cohorts tracheal aspirate samples. The lowest soft-thresholding power that achieved a scale-free topology was used. In consensus module analysis of BAL and tracheal aspirate samples the recommended default of 12 was used as filtering thresholds had competing impacts on the optimal power. Signed networks were constructed in a single block with a minimum module size of 30 and dynamic tree cutting. Modules with a cut height <0.25 were merged. Module membership was calculated as the Pearson correlation between the normalized count and the module eigengene with FDR correction.

#### Pathway analysis

Pathway enrichment was performed on differentially expressed genes and WGCNA module hub genes (defined as absolute module membership >0.8) through g:Profiler R client^73^. For WGCNA modules, Over Representation Analysis (ORA) was performed against Gene Ontology Biological Processes (excluding inferred from electronic annotation evidence codes), Reactome and TRANSFAC databases using a custom background with a significance threshold <0.05 after g:SCS correction. Unlike Benjamini-Hochberg FDR correction, the g:SCS algorithm does not assume test independence, an assumption necessarily violated by hierarchical gene ontology terms. Geneset Enrichment Analysis (GSEA) was performed on differentially expressed genes using ClusterProfiler R package ranked by log-fold change, with Benjamini-Hochberg correction (g:SCS not available for GSEA) and a significance threshold of <0.05. Network plots were constructed from significantly enriched pathways and their core enriched genes (or overlapping genes for ORA). Protein-Protein Interactions between core enriched genes with >80% confidence from StringDB (strind-db.org, version 12) were incorporated into the network as edges.

Upstream transcription factor prediction was performed using Transcription Factor Enrichment Analysis using ChEA3^25^. This analysis was conducted separately for positively and negatively differentially expressed genes for each Pneumotype as differential expression on clusters produces complementary gene lists – with each upregulated gene appearing downregulated in another cluster.

#### Survival and time to extubation analysis

Survival was assessed with censoring at 1 year using a Cox proportional hazards regression model including Pneumotype and age (HR 1.03, *p* = 0.036). Time to extubation was assessed using Pneumotype alone as age had no effect in this model (HR 1.00, *p* = 0.7). Severity of illness and respiratory failure were not included as it is hypothesized that Pneumotype may have causal influence on these. Kaplan-Meier curves where also fit (Fig. 1F, G). Analysis was performed using the survival R package.

#### Inflammatory proteins

Measured inflammatory proteins (pg/ml) were not normally distributed and showed significant skew. Differences between Pneumotypes were assessed using Kruskal Wallis Rank Sum and *P* values were FDR adjusted for 96 comparisons (48 BAL and 48 plasma). Post hoc pairwise comparisons was by Dunn’s test. Pearson Correlation Coefficients were calculated on log1p transformed values and P values calculated using Student’s asymptotic *p* value for correlation then FDR adjusted for 48×48 comparisons. Following log1p transformation skewness was not <|2| for plasma SDF-1a, IL-9, MIP-1a and BAL MIF, GM-CSF and IL-13 and correlation should be interpreted with caution for these proteins. Scatter plots for highly correlated inflammatory proteins were inspected to assess the influence of outliers.

#### Validation

No equivalent cohort of suspected adult pneumonia patients with whole BAL RNAseq was identified, however Wauters et al.^48^ included single cell sequencing from BAL samples from 35 COVID-19 and pneumonia controls, though not all were mechanically ventilated. Grant et al.^49^ included RNAseq of flow-sorted macrophages and flow cytometry from BAL in patients with microbiologically confirmed pneumonia (and non-pneumonia ICU controls) as a comparator to COVID-19 pneumonia. Additionally Langelier et al.^21^ have made tracheal aspirate RNAseq and clinical data available for a closely related clinical cohort of ventilated patients being investigated for suspected pneumonia.

Analysis of the Wauters^48^ dataset was facilitated by collaboration with the original group through a data sharing agreement. Pseudobulking was performed using the Seurat package v5^74^, aggregating raw counts per patient followed by VST normalisation. Downstream clustering, differential expression analysis and comparison of clinical characteristics were performed as described for the discovery cohort above. Correlation of normalised counts with the WGCNA-derived modules from the discovery cohort was undertaken for each W-cluster by Pearson’s correlation coefficient. Single cell labels from the original manuscript^48^ were utilised for comparison of major cell types. Re-labelling was required to undertake the exploratory evaluation for distal alveolar stem cell (DASC) and neutrophil immaturity hypotheses. Initial filtering thresholds for cells and genes were set as per original analysis. This included minimum unique genes (151), unique molecular identifiers (UMI) (301) and proportion of mitochondrial RNA (mtRNA) reads per cell ( < 20%), and the removal of B and T cell receptor, immunoglobin and haemoglobin genes from highly variable genes^48^. Counts were log normalised using Seurat’s NormaliseData function. Additionally, S and G2M cell cycle phase scores, mtRNA, UMIs, and patient IDs were variables scaled for before 3000 most highly variable genes were used for PCA clustering. Doublets were removed using scDblFinder^75^ per patient, informed by expected doublet rates according to the number of cells from the manufacturer’s instructions (10X Genomics, CA, USA). Batch correction was undertaken using Harmony integration within Seurat^76^. A resolution of 0.8 for finding neighbours using the Louvain algorithm, and 20 principal components were chosen for final clustering. Seurat’s AddModuleScore function was used to compare marker gene signatures against 100 control genes and the highest AddModuleScore for cell type assignment. DASCs were annotated by mandatory expression of both stem genes *SOX2, SOX9*, alongside established DASC signature genes *KRT5, TP63,GSTA2, GSTA1, LMO3, PPARGC1A, RPS15A, ALDH1A1, SCGB1A1, TF, GOLGA8A, ATP5MG*^44–46^ and neutrophil subtypes identified within the neutrophil population defined in the original manuscript, using subtype signatures defined by Kwok et al.^26^ and expressed as a percentage of cells in a patient sample.

In the Grant cohort bulk RNAseq was limited to flow-sorted macrophages and thus any epithelial cell transcription defining Pn2 could not be assessed. However, this data clustered on M1/M2 macrophage polarization, and the flow cytometry data for these samples could be used to validate the predicted cellularity associated with this in Pn1 and Pn3.

Comparisons of tracheal aspirate and bronchoalveolar lavage host transcriptomes is not well described in the literature, and the impact of differing sampling methods is unknown. However, we attempted to replicate our clustering findings of enrichment for bacterial organisms and immunosuppression. Significance testing of these proportions was tested by Pearson’s Chi-squared test. WGCNA consensus module analysis was used to compare gene modules common to the BAL and TA datasets and thus similarities and differences in expression between clusters and datasets. As tracheal aspirates are less likely to sample alveolar cells, this effect was estimated using xCell deconvolution on the pooled BAL and TA data.

## Supplementary information

Supplementary Information

Description of Additional Supplementary Files

Supplementary Data 1

Transparent Peer Review file

## Source data

Source Data

## Data Availability

Supplemental Data File 1 and Source Data for the generation of this manuscript are available at 10.6084/m9.figshare.27029002. Supplemental Data File 1 contains abbreviated clinical meta-data, full clinical meta-data are under restricted access due to ethical review board permissions but can be obtained from the corresponding author following provision of a data sharing agreement. Raw sequencing data for RNA has been deposited in the European Nucleotide Archive, deposition reference EGAS00001003074. For Wauters data, raw sequencing reads of the scRNA-seq are deposited in the EGA European Genome-Phenome Archive database (EGAS00001004717). A download of the read count matrix is available at Lambrecht’s lab immune atlas. Clinical metadata was available by data transfer agreement that precludes public deposition, but is available on request to the authors of Wauter’s paper. For Grant^49^ Raw data are available through the dbGaP repository (accession phs002300.v2.p1) and Bulk RNA-seq counts tables and metadata are included as supplementary dataset 2 and dataset 3 whilst flow cytometry data is included in supplementary dataset 9 in the original manuscript^49^. For Langellier host transcript counts are tabulated in dataset S09, clinical metadata in dataset S01, microbiology in dataset S03 in the original paper^21^. All other data are available in the article and its Supplementary files or from the corresponding author upon request. Source data are provided with this paper.

## Code availability

No new code was written for this manuscript, however the code used for analyses are viewable at https://gitfront.io/r/mark-jeffrey/eZYpfcdwm7Eu/vapR/
