# Supplementary material for: Pulmonary inflammation in severe pneumonia is characterised by compartmentalised and mechanistically distinct sub-phenotypes
Source: Nat Commun. 2026 Jun 23;17:5312. doi: 10.1038/s41467-026-74190-x (PMC13291267; doi:10.1038/s41467-026-74190-x)
Supplement: Supplementary file 1 — Supplementary Information [file 41467_2026_74190_MOESM1_ESM.pdf]

Supplementary Table 1: Broncho-Alveolar Lavage inflammatory protein panel results by Pneumotype. All values pg/ml. P-values by two-sided Kruskal-Wallis one way analysis of variance, q-value Benjamini-Hochberg adjusted values for multiplicity.

BAL Cytokines (pg/ml)	Overall N = 80	Pn1 N = 39	Pn2 N = 19	Pn3 N = 22	p-value	q-value
Basic FGF	11 (6-19)	10 (4-15)	12 (6-17)	20 (11-29)	<0.001	0.003
CCL11	4 (1-12)	7 (2-14)	2 (1-5)	4 (1-16)	0.2	0.2
CCL2	563 (127-1,059)	611 (121-1,187)	344 (56-864)	632 (133-1,003)	0.8	0.8
CCL27	11 (6-19)	9 (4-22)	11 (6-15)	12 (7-22)	0.7	0.7
CCL3	4 (1-23)	2 (1-4)	7 (3-25)	25 (4-211)	<0.001	<0.001
CCL4	33 (9-110)	18 (5-44)	34 (8-99)	257 (57-791)	<0.001	<0.001
CCL5	12 (6-21)	10 (5-18)	12 (6-43)	16 (11-30)	0.3	0.3
CCL7	12 (3-38)	11 (3-29)	11 (2-17)	17 (5-55)	0.5	0.6
CLEC11A	3,718 (1,221-6,738)	3,725 (2,416-8,065)	2,999 (145-6,091)	4,011 (1,521-4,902)	0.6	0.7
CXCL1	3,104 (1,419-6,487)	2,171 (1,002-3,548)	4,779 (1,600-15,083)	4,099 (2,821-13,017)	0.002	0.005
CXCL12	44 (23-129)	80 (23-183)	34 (19-79)	45 (26-79)	0.4	0.4
CXCL8	460 (168-2,340)	215 (135-473)	645 (199-3,280)	3,585 (1,166-10,770)	<0.001	<0.001
CXCL9	311 (73-1,725)	161 (33-736)	504 (43-4,302)	441 (141-4,480)	0.071	0.10
G-CSF	270 (96-629)	148 (46-339)	339 (97-586)	758 (312-3,247)	<0.001	<0.001
GM-CSF	1.63 (0.84-3.02)	1.62 (0.60-2.85)	1.59 (1.03-2.45)	2.77 (1.34-3.68)	0.2	0.3
HGF	162 (67-336)	164 (60-464)	126 (80-257)	181 (129-447)	0.5	0.6
IFN-a2	7 (4-12)	6 (4-10)	8 (5-11)	11 (7-14)	0.028	0.043
IFN-g	22 (5-72)	20 (4-71)	10 (5-51)	36 (12-109)	0.079	0.11
IL-10	7 (5-12)	6 (4-9)	6 (4-11)	11 (6-31)	0.047	0.071
IL-12 (p40)	59 (15-133)	41 (9-112)	42 (15-100)	88 (60-213)	0.008	0.015
IL-12(p70)	2.73 (1.51-3.92)	2.09 (1.07-3.33)	2.84 (1.80-3.66)	3.68 (2.09-5.25)	0.006	0.013
IL-13	0.74 (0.51-1.20)	0.62 (0.46-0.96)	0.84 (0.51-1.14)	0.96 (0.45-1.43)	0.3	0.4
IL-16	61 (35-107)	39 (27-59)	97 (33-150)	110 (79-166)	<0.001	<0.001
IL-17	8 (4-14)	5 (4-10)	8 (5-15)	10 (7-34)	0.008	0.015
IL-18	10 (6-19)	10 (4-14)	8 (6-21)	16 (8-35)	0.028	0.043
IL-1a	10 (7-22)	8 (6-11)	12 (5-18)	20 (11-44)	0.001	0.004
IL-1b	5 (2-18)	2 (2-5)	9 (3-32)	32 (6-248)	<0.001	<0.001
IL-1ra	908 (285-1,964)	475 (172-1,160)	562 (243-2,585)	1,832 (1,179-3,362)	<0.001	<0.001
IL-2	3.1 (2.2-7.1)	2.4 (1.6-4.3)	3.5 (2.2-6.7)	7.3 (3.1-13.1)	<0.001	0.001
IL-2Ra	15 (9-29)	12 (9-23)	13 (7-28)	21 (15-40)	0.12	0.2
IL-3	0.55 (0.30-0.85)	0.44 (0.25-0.64)	0.51 (0.39-0.63)	0.92 (0.53-1.14)	0.002	0.006
IL-4	1.09 (0.70-2.30)	0.84 (0.63-1.65)	1.07 (0.72-1.91)	2.23 (1.24-3.38)	0.013	0.023
IL-5	23 (6-42)	16 (5-39)	14 (6-38)	33 (17-61)	0.026	0.043
IL-6	114 (22-417)	37 (15-328)	142 (37-248)	252 (101-1,157)	0.024	0.041
IL-7	14 (8-33)	12 (7-21)	21 (9-31)	15 (7-41)	0.7	0.7
IL-9	14 (9-27)	12 (7-19)	12 (9-20)	24 (18-43)	0.002	0.006

IP-10	712 (141-2,700)	1,011 (227-4,146)	369 (134-3,262)	1,412 (99-2,551)	0.7	0.7
Il-15	62 (17-177)	35 (7-169)	44 (19-161)	163 (64-235)	0.004	0.010
KITLG	21 (11-43)	22 (11-43)	21 (14-46)	20 (12-40)	>0.9	>0.9
LIF	28 (14-60)	23 (7-39)	22 (7-43)	66 (37-159)	<0.001	<0.001
LTA	6 (2-12)	4 (1-9)	4 (3-9)	10 (7-26)	<0.001	0.003
M-CSF	10 (5-28)	7 (4-12)	10 (5-35)	18 (11-53)	0.008	0.015
MIF	1,617 (921-3,088)	1,187 (780-2,140)	1,963 (1,486-3,237)	2,753 (1,298-3,838)	0.005	0.011
NGF	1.74 (0.77-3.03)	1.23 (0.60-2.52)	1.26 (0.69-2.86)	3.00 (1.66-5.26)	0.004	0.009
PDGF-BB	35 (21-56)	30 (17-46)	34 (25-46)	53 (43-66)	0.011	0.020
TNF-a	15 (7-40)	9 (6-17)	17 (7-29)	53 (20-222)	<0.001	<0.001
TNFSF10	43 (15-144)	27 (12-55)	71 (22-209)	140 (50-330)	<0.001	<0.001
VEGFA	41 (1-198)	6 (1-110)	42 (1-131)	205 (42-263)	<0.001	<0.001

Supplementary Table 2: Serum inflammatory protein panel results by Pneumotype. All values pg/ml. All values pg/ml. P value by two sided Kruskal-Wallis one way analysis of variance, q-value Benjamini-Hochberg adjusted values for multiplicity.

Serum Cytokines (pg/ml)	Overall N = 79	Pn1 N = 38	Pn2 N = 19	Pn3 N = 22	p-value	q-value
Basic FGF	25 (21-29)	26 (23-30)	21 (19-25)	23 (21-30)	0.050	0.6
CCL11	9 (7-15)	9 (6-17)	13 (8-15)	8 (6-13)	0.3	0.7
CCL2	17 (11-46)	17 (11-35)	16 (12-46)	20 (11-67)	0.9	>0.9
CCL27	102 (64-161)	103 (64-146)	92 (64-188)	101 (57-161)	>0.9	>0.9
CCL3	1.39 (0.83-2.50)	1.14 (0.92-2.51)	1.25 (0.81-2.32)	1.50 (0.94-2.62)	0.7	>0.9
CCL4	24 (20-28)	24 (20-28)	22 (20-27)	26 (17-31)	0.8	>0.9
CCL5	872 (307-1,704)	649 (219-1,483)	1,258 (502-1,927)	716 (435-1,619)	0.2	0.6
CCL7	3.15 (1.55-4.91)	3.37 (2.28-4.92)	1.90 (0.59-4.19)	3.23 (0.81-4.72)	0.2	0.6
CLEC11A	25,588 (14,615-40,991)	21,967 (14,615-38,246)	25,857 (12,473-40,917)	29,890 (15,210-44,714)	0.9	>0.9
CXCL1	87 (73-391)	82 (73-296)	85 (72-660)	151 (73-423)	0.4	0.8
CXCL12	155 (106-198)	160 (112-193)	155 (102-242)	147 (106-213)	>0.9	>0.9
CXCL8	15 (7-25)	15 (7-25)	9 (7-22)	15 (8-28)	0.8	>0.9
CXCL9	65 (36-185)	60 (34-127)	66 (32-210)	66 (42-187)	0.8	>0.9
G-CSF	54 (32-119)	51 (33-115)	49 (23-81)	70 (35-126)	0.7	>0.9
GM-CSF	1.62 (0.48-2.69)	2.12 (0.49-3.37)	1.42 (0.10-2.09)	1.26 (0.48-2.12)	0.2	0.6
HGF	373 (256-700)	371 (271-624)	350 (176-570)	485 (304-767)	0.2	0.6
IFN-a2	6.9 (5.7-9.7)	7.8 (6.2-10.2)	6.9 (5.7-8.3)	6.7 (4.8-9.5)	0.5	0.8
IFN-g	11 (6-20)	11 (7-18)	9 (5-14)	13 (7-43)	0.2	0.6
IL-10	2 (0-10)	3 (0-11)	1 (0-4)	4 (1-10)	0.040	0.6
IL-12 (p40)	15 (6-80)	16 (6-80)	15 (6-30)	17 (15-93)	0.2	0.6
IL-12(p70)	1.68 (0.37-3.86)	1.91 (0.37-4.20)	1.45 (0.37-3.05)	1.51 (0.75-2.73)	0.7	>0.9
IL-13	2.73 (1.53-3.72)	3.06 (1.61-4.05)	3.06 (1.61-4.08)	2.36 (1.08-3.06)	0.090	0.6
IL-16	41 (7-56)	49 (30-68)	36 (4-53)	43 (2-53)	0.3	0.7
IL-17	4.6 (2.0-7.3)	4.3 (2.4-7.3)	4.6 (0.8-6.4)	4.4 (2.5-7.7)	0.6	>0.9
IL-18	41 (22-86)	50 (28-124)	34 (17-62)	37 (14-72)	0.2	0.6
IL-1a	4.4 (0.7-6.8)	4.2 (0.7-8.0)	2.2 (0.7-6.0)	4.9 (0.7-6.2)	0.5	0.8
IL-1b	3.34 (2.36-4.40)	3.55 (2.43-3.91)	3.29 (2.43-4.40)	3.17 (2.13-4.64)	>0.9	>0.9
IL-1ra	19 (19-220)	19 (19-84)	19 (19-289)	156 (19-1,709)	0.031	0.6
IL-2	3.3 (0.2-7.6)	5.7 (1.3-10.7)	2.4 (0.2-4.2)	2.9 (1.6-6.8)	0.039	0.6
IL-2Ra	60 (37-118)	67 (47-102)	45 (31-90)	58 (42-147)	0.4	0.8
IL-3	0.38 (0.01-0.63)	0.32 (0.01-0.69)	0.38 (0.01-0.54)	0.34 (0.12-0.57)	0.7	>0.9
IL-4	0.75 (0.10-1.12)	0.68 (0.10-1.24)	0.83 (0.05-1.11)	0.75 (0.28-1.08)	0.6	>0.9
IL-5	5 (3-26)	9 (3-33)	5 (2-20)	5 (4-22)	0.2	0.6
IL-6	17 (7-38)	12 (7-27)	14 (5-31)	26 (9-62)	0.2	0.6
IL-7	19 (10-28)	22 (12-28)	16 (9-28)	22 (9-25)	0.4	0.8
IL-9	29 (23-45)	30 (23-43)	28 (24-45)	31 (23-46)	>0.9	>0.9
IP-10	186 (92-556)	180 (92-534)	155 (90-339)	251 (101-670)	0.6	>0.9

Il-15	13 (4-42)	13 (4-67)	13 (4-13)	13 (4-42)	0.7	>0.9
KITLG	30 (15-45)	34 (21-48)	18 (13-38)	31 (15-43)	0.12	0.6
LIF	32 (22-48)	44 (26-56)	32 (9-37)	30 (17-39)	0.080	0.6
LTA	0 (0-33)	0 (0-31)	0 (0-33)	1 (0-36)	0.6	>0.9
M-CSF	31 (18-46)	34 (26-57)	20 (14-36)	29 (17-45)	0.10	0.6
MIF	238 (161-352)	249 (172-440)	182 (144-288)	243 (157-409)	0.3	0.7
NGF	0.84 (0.45-1.46)	1.09 (0.56-1.49)	0.66 (0.06-1.07)	0.69 (0.47-1.49)	0.060	0.6
PDGF-BB	162 (96-247)	141 (68-204)	202 (115-304)	169 (122-297)	0.2	0.6
TNF-a	26 (15-34)	27 (19-36)	22 (12-30)	27 (13-33)	0.2	0.6
TNFSF10	11 (7-14)	10 (7-12)	11 (8-13)	12 (6-17)	0.6	>0.9
VEGFA	38 (6-64)	37 (4-66)	32 (6-54)	40 (6-57)	0.9	>0.9

Supplementary Table 3: Clinical and demographic features of the patients when clustered by blood type (Blood 1 (B1) or Blood (B2) or SRS type (SRS1-3)[15]. Continuous values shown as median and interquartile range, categorical by n (%). P-value by Chi^2 for categorical data or two-sided Fisher's exact when values were <5. P-value by Wilcoxon rank sum for continuous variables for blood type, and two-sided Kruskal-Wallis one way analysis of variance for SRS type.

Group		Overall N = 74	B1 N = 18	B2 N = 56	p-value	Overall N = 74	SRS1 N = 21	SRS2 N = 44	SRS3 N = 9	p-value
Demographics	Age (Years)	60 (44, 70)	60 (50, 78)	60 (43, 70)	0.7	60 (44, 70)	61 (53, 75)	60 (43, 70)	43 (41, 54)	0.029
	Female	31 (42%)	7 (39%)	24 (43%)	0.8	31 (42%)	11 (52%)	16 (36%)	4 (44%)	0.5
	BMI >30kg/m^2^	15 (20%)	4 (22%)	11 (20%)	>0.9	15 (20%)	4 (19%)	9 (20%)	2 (22%)	>0.9
	Immunosuppressed	30 (41%)	10 (56%)	20 (36%)	0.14	30 (41%)	10 (48%)	17 (39%)	3 (33%)	0.7
	Neutropenic	5 (6.8%)	3 (17%)	2 (3.6%)	0.089	5 (6.8%)	2 (9.5%)	2 (4.5%)	1 (11%)	0.5
	Transplant recipient	5 (6.8%)	2 (11%)	3 (5.4%)	0.6	5 (6.8%)	3 (14%)	0 (0%)	2 (22%)	0.006
	Admission APACHE II	16 (12, 22)	23 (20, 27)	14 (11, 19)	<0.001	16 (12, 22)	21 (17, 27)	15 (12, 19)	12 (10, 15)	0.002
	PF ratio	23 (17, 31)	20 (13, 25)	24 (17, 33)	0.2	23 (17, 31)	20 (13, 26)	24 (18, 33)	24 (17, 31)	0.3
	ARDS	42 (58%)	12 (71%)	30 (55%)	0.2	42 (58%)	13 (62%)	24 (57%)	5 (56%)	>0.9
	Shock	15 (20%)	6 (33%)	9 (16%)	0.2	15 (20%)	4 (19%)	10 (23%)	1 (11%)	>0.9
	Illness onset prior to BAL (Days)	-1.5 (-5.0, -1.0)	-2.5 (-3.0, -1.0)	-1.0 (-6.0, 0.0)	0.3	-1.5 (-5.0, -1.0)	-1.0 (-3.0, -1.0)	-1.0 (-6.0, -1.0)	-2.0 (-8.0, -1.0)	0.5
	Antibiotic free days (Day 28)	10 (2, 20)	3 (0, 15)	12 (5, 20)	0.018	10 (2, 20)	7 (0, 18)	11 (5, 20)	15 (3, 19)	0.3
	In-Hospital Mortality	24 (32%)	10 (56%)	14 (25%)	0.016	24 (32%)	8 (38%)	14 (32%)	2 (22%)	0.8
	Ventilator free days (Day 28)	6 (0, 21)	0 (0, 24)	8 (0, 21)	0.4	6 (0, 21)	6 (0, 24)	8 (0, 21)	1 (0, 9)	0.6
Blood results	White cell count (x10^9^/L)	11 (6, 19)	16 (5, 24)	10 (7, 16)	0.5	11 (6, 19)	18 (6, 24)	10 (7, 16)	10 (6, 11)	0.3
	Neutrophil count(x10^9^/L)	9 (5, 17)	15 (4, 23)	8 (6, 13)	0.3	9 (5, 17)	17 (5, 23)	8 (6, 14)	8 (4, 9)	0.069
	Lactate (mmol/L)	1.30 (1.00, 2.00)	1.70 (1.40, 2.10)	1.20 (0.95, 1.85)	0.005	1.30 (1.00, 2.00)	1.70 (1.40, 1.90)	1.30 (1.00, 2.20)	1.00 (0.80, 1.30)	0.053
	CRP (mg/L)	187 (126, 274)	300 (246, 330)	150 (75, 232)	<0.001	187 (126, 274)	274 (206, 318)	170 (134, 238)	120 (94, 158)	0.004
Evidence	Radiological evidence of pneumonia	57 (77%)	17 (94%)	40 (71%)	0.055	57 (77%)	20 (95%)	31 (70%)	6 (67%)	0.037

	Microbiological evidence of pneumonia	37 (50%)	11 (61%)	26 (46%)	0.3	37 (50%)	10 (48%)	23 (52%)	4 (44%)	0.9
Aetiology of adjudicated pneumonia	Cases adjudicated as pneumonia	30 (41%)	13 (72%)	17 (30%)	0.002	30 (41%)	12 (57%)	17 (39%)	1 (11%)	0.069
	Bacteria pneumonia	19 (26%)	8 (44%)	11 (20%)	0.060	19 (26%)	9 (43%)	10 (23%)	0 (0%)	0.037
	Viral pneumonia	10 (14%)	4 (22%)	6 (11%)	0.2	10 (14%)	2 (9.5%)	7 (16%)	1 (11%)	0.9
	Fungal pneumonia	3 (4.1%)	3 (17%)	0 (0%)	0.013	3 (4.1%)	3 (14%)	0 (0%)	0 (0%)	0.033
Blood quality	Library storage time (days)	333 (170, 453)	376 (201, 474)	329 (106, 434)	0.3	333 (170, 453)	334 (201, 476)	313 (106, 440)	359 (200, 402)	0.6
Admission diagnosis	Admission diagnosis				0.12					0.2
	Non-infectious non-respiratory organ failure	16 (22%)	2 (11%)	14 (25%)		16 (22%)	2 (9.5%)	12 (27%)	2 (22%)	
	Non-infectious Respiratory Failure	16 (22%)	2 (11%)	14 (25%)		16 (22%)	4 (19%)	9 (20%)	3 (33%)	
	Non-pulmonary Infection	7 (9.5%)	4 (22%)	3 (5.4%)		7 (9.5%)	3 (14%)	3 (6.8%)	1 (11%)	
	Pulmonary Infection	32 (43%)	9 (50%)	23 (41%)		32 (43%)	9 (43%)	20 (45%)	3 (33%)	
	Transplant	3 (4.1%)	1 (5.6%)	2 (3.6%)		3 (4.1%)	3 (14%)	0 (0%)	0 (0%)	

Supplementary Table 4: Clinical and demographic features of patients from Wauters et al[48] clustered by pseudobulked single cell RNA sequencing into W1, W2 and W3. Continuous values shown as median and interquartile range, categorical by n (%). P-value by Chi^2 for categorical data. P-value by two-sided Kruskal-Wallis one way analysis of variance for continuous variables.

**Wauters et al BAL Clusters**	**Overall,** N = 35	N = 13, **W1**	N = 8, **W2**	N = 14, **W3**	**p-value**
Age	67.00 [56.00-71.00]	68.00 [61.00-71.00]	71.50 [60.00-73.00]	56.00 [49.25-66.50]	0.0116
Female	12 (34.3%)	6 (46.2%)	4 (50.0%)	2 (14.3%)	0.124
BMI	27.55 [24.64-29.64]	25.20 [22.51-28.73]	28.91 [25.91-30.58]	27.58 [27.21-28.86]	0.323
Type of Patient					0.0248
ICU - Mechanical Ventilation	21 (60.0%)	4 (30.8%)	6 (75.0%)	11 (78.6%)	
Ward	14 (40.0%)	9 (69.2%)	2 (25.0%)	3 (21.4%)	
Days of Invasive Ventilation	10.00 [0.00-23.00]	0.00 [0.00-11.00]	14.25 ± 14.66	18.25 [5.75-34.50]	0.0994
ICU Mortality	2 (5.7%)	0 (0.0%)	1 (12.5%)	1 (7.1%)	0.466
Hospital Mortality	4 (11.4%)	1 (7.7%)	1 (12.5%)	2 (14.3%)	0.86
Length of Stay - ICU	16.00 [0.00-32.50]	0.00 [0.00-16.00]	20.00 [10.75-27.25]	27.00 [12.25-52.75]	0.0494
Length of Stay - Hospital	39.00 [18.50-56.50]	15.00 [11.00-39.00]	41.00 [37.25-44.25]	42.00 [29.00-81.75]	0.0586
White Blood Cells (x10^9L)	9.71 [6.12-11.57]	9.04 [5.95-11.73]	9.66 [4.30-10.81]	10.34 [7.30-11.79]	0.574
Neutrophils (x10^9L)	7.65 [4.08-9.57]	7.35 [3.77-9.85]	7.25 [2.70-9.12]	7.95 [5.22-8.93]	0.749
C-Reactive Protein (mg/L)	94.65 [32.42-192.32]	67.95 [25.83-178.82]	100.45 [24.88-150.62]	125.35 [53.85-233.60]	0.369
Immunosuppressed	11 (31.4%)	6 (46.2%)	2 (25.0%)	3 (21.4%)	0.348
COVID-19 Patients	22 (62.9%)	5 (38.5%)	5 (62.5%)	12 (85.7%)	0.0398
Viral Pathogen	23 (65.7%)	5 (38.5%)	6 (75.0%)	12 (85.7%)	0.0291
Bacterial Pathogen	13 (37.1%)	4 (30.8%)	2 (25.0%)	7 (50.0%)	0.423
Fungal Pathogen	7 (20.0%)	3 (23.1%)	1 (12.5%)	3 (21.4%)	0.829
No Pathogenic Organism	5 (14.3%)	2 (15.4%)	2 (25.0%)	1 (12.5%)	0.51
No Organism	4 (11.4%)	2 (15.4%)	1 (12.5%)	1 (7.1%)	0.793
Type of Pneumonia					0.058
Community acquired pneumonia	10 (28.6%)	7 (53.8%)	0 (0.0%)	3 (21.4%)	
Hospital acquired pneumonia	5 (14.3%)	2 (15.4%)	2 (25.0%)	1 (7.1%)	
Ventilator acquired pneumonia	20 (57.1%)	4 (30.8%)	6 (75.0%)	10 (71.4%)	

Supplementary Table 5: Clinical and demographic and cellular features of patients from Grant et al[49] clustered by sorted alveolar macrophage bulk RNA sequencing into Grant_1, Grant_2 and Grant_3. Continuous values shown as median and interquartile range, categorical by n (%). P-value by Chi^2 for categorical data or two-sided Fisher's exact when values were <5. P-value by two-sided Kruskal-Wallis one way analysis of variance for continuous variables.

**Characteristic**	**Overall**<br>**N = 167**	**Grant 1**<br>**N = 59**	**Grant 2**<br>**N = 78**	**Grant 3**<br>**N = 30**	**p-value**
Age	60 (47, 70)	60 (43, 70)	57 (44, 68)	69 (60, 74)	0.002
Female	69 (41%)	25 (42%)	38 (49%)	6 (20%)	0.025
Days since intubation	3 (1, 11)	6 (1, 18)	3 (1, 8)	2 (1, 9)	0.073
Diagnosis					
Bacterial Pneumonia	79 (47%)	22 (37%)	33 (42%)	24 (80%)	
COVID-19	45 (27%)	12 (20%)	32 (41%)	1 (3.3%)	
Non-Pneumonia Control	21 (13%)	14 (24%)	6 (7.7%)	1 (3.3%)	
Viral Pneumonia	22 (13%)	11 (19%)	7 (9.0%)	4 (13%)	
Bacterial Superinfection (if Viral)					0.007
Not Viral	102 (61%)	37 (63%)	39 (50%)	26 (87%)	
Primary Only	48 (29%)	16 (27%)	30 (38%)	2 (6.7%)	
Superinfection	17 (10%)	6 (10%)	9 (12%)	2 (6.7%)	
CD206 high Macs (% Macs)	56 (33, 73)	73 (60, 82)	51 (33, 64)	24 (14, 41)	<0.001
CD206 low Macs (% Macs)	44 (27, 67)	27 (18, 40)	49 (36, 67)	76 (59, 86)	<0.001
CD3+ T-cells (% total)	5 (1, 14)	5 (2, 14)	7 (3, 22)	1 (0, 2)	<0.001
CD4+ T-cells (% total)	2 (0, 6)	2 (0, 6)	3 (1, 9)	0 (0, 1)	<0.001
CD8+ T-cells (% total)	2 (0, 6)	2 (1, 5)	4 (1, 11)	0 (0, 1)	<0.001
Neutrophils (% total)	53 (21, 82)	35 (14, 63)	50 (20, 70)	94 (84, 96)	<0.001
Macrophages (% total)	27 (7, 48)	42 (22, 69)	24 (10, 39)	2 (1, 9)	<0.001
Monocytes (% total)	4.1 (2.2, 7.4)	3.4 (1.1, 6.7)	5.9 (3.1, 10.0)	3.1 (2.1, 4.1)	<0.001

Supplementary Table 6: Clinical and demographic features of patients from Langellier et al[21] clustered by tracheal aspirate bulk RNA sequencing into TA1 and TA2. Continuous values shown as median and interquartile range, categorical by n (%). P-value by Chi^2 for categorical data or two-sided Fisher's exact when values were <5. P-value by two-sided Wilcoxon rank sum for continuous variables.

**Langelier _et al_ Tracheal Aspirate Clusters**	**Overall**<br>**N = 84**	**TA1**<br>**N = 35**	**TA2**<br>**N = 49**	**p-value**
Age (years)	64 (54, 74)	66 (58, 76)	63 (54, 73)	0.4
Female	28 (33%)	14 (40%)	14 (29%)	0.3
White Blood Cell Max (10^9/ml)	14 (10, 18)	14 (9, 18)	14 (11, 19)	0.8
Immunosuppressed	36 (43%)	17 (49%)	19 (39%)	0.4
Systemic Inflammation	81 (96%)	32 (91%)	49 (100%)	0.069
Viral Pathogen	15 (18%)	9 (26%)	6 (12%)	0.11
Bacterial Pathogen	49 (58%)	16 (46%)	33 (67%)	0.047
Adjudicated Pneumonia	55 (65%)	19 (54%)	36 (73%)	0.068
Type of Pneumonia				0.6
Community acquired pneumonia	11 (20%)	4 (22%)	7 (19%)	
Community acquired pneumonia with healthcare exposure	28 (52%)	11 (61%)	17 (47%)	
Hospital acquired pneumonia	12 (22%)	3 (17%)	9 (25%)	
Ventilator associated pneumonia	3 (5.6%)	0 (0%)	3 (8.3%)	

Supplementary Figure 1: clustering metrics for gene expression. A Elbow plot for BAL demonstrating an elbow at 3 and no further reduction in with-group sum of squares between 3 and 5 clusters. **B** Silhouette score plot for BAL with maximal scores between two and three clusters. **C** Gap statistic plot for BAL indicating a local maximum at three clusters. **D-E** Elbow, Silhouette score and Gap statistic plots for Blood gene expression. **G-I** Elbow, Silhouette score and Gap statistic plots for pseudobulked scRNAseq data from Wauters et al [48]. **J-L** Elbow, Silhouette score and Gap statistic plots for sorted macrophage bulk RNA data from Grant et al [49]. **M-O** Elbow, Silhouette score and Gap statistic plots for bulk RNA from Langellier et al [21].

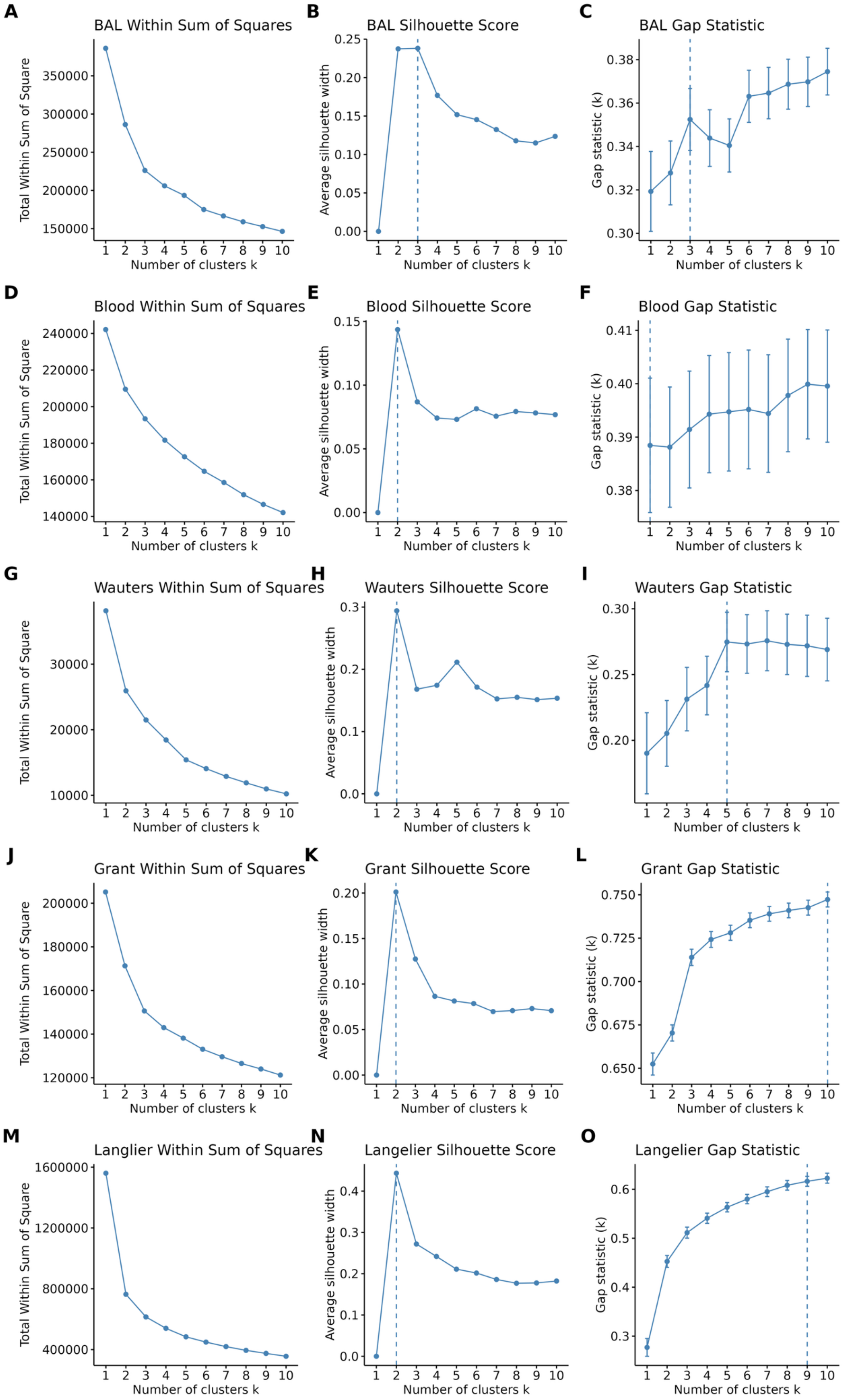
A
BAL Within Sum of Squares
B
BAL Silhouette Score
C
BAL Gap Statistic
D
Blood Within Sum of Squares
E
Blood Silhouette Score
F
Blood Gap Statistic
G
Wauters Within Sum of Squares
H
Wauters Silhouette Score
I
Wauters Gap Statistic
J
Grant Within Sum of Squares
K
Grant Silhouette Score
L
Grant Gap Statistic
M
Langlier Within Sum of Squares
N
Langelier Silhouette Score
O
Langelier Gap Statistic
Total Within Sum of Square
Average silhouette width
Gap statistic (k)
Number of clusters k

Supplementary Figure 2: RNAsep library quality control

Clustering was not driven by library size or number of non-zero genes (nFeatures) coloured by Pneumotype.

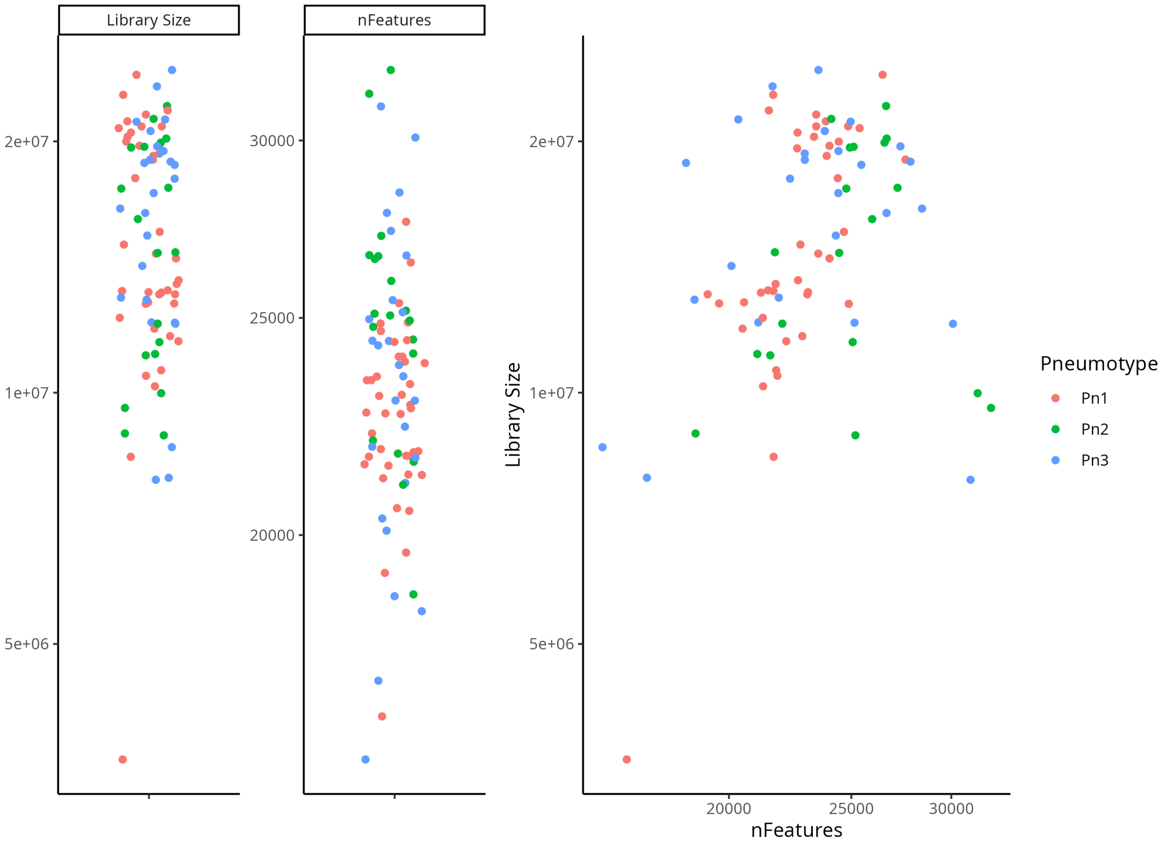

Supplementary Figure 3: Relative indifference of pneumotype to temporal factors. **A** Boxplot of time interval from illness onset to time of BAL. N=39 Pn1, 19 Pn2 and 22 Pn3. P values by two-sided Wilcoxon rank sum test. **B** Principal component analysis plot of the bronchoalveolar transcriptional clusters highlighting the four patients who were resampled. Arrows link the first and second resampling, with the gap in days between the samples indicated, with pneumotype at time of sampling indicated by colour (Pn1 red, Pn2 green, Pn3 blue). For the patients resampled at 5, 11 and 13 days these were during the same admission and same episode of pneumonia, with resampling prompted by a deterioration in clinical picture and suspicion of recurrent infection. The patient sampled 43 days apart occurred on two separate ICU admissions with distinct episodes of suspected pneumonia

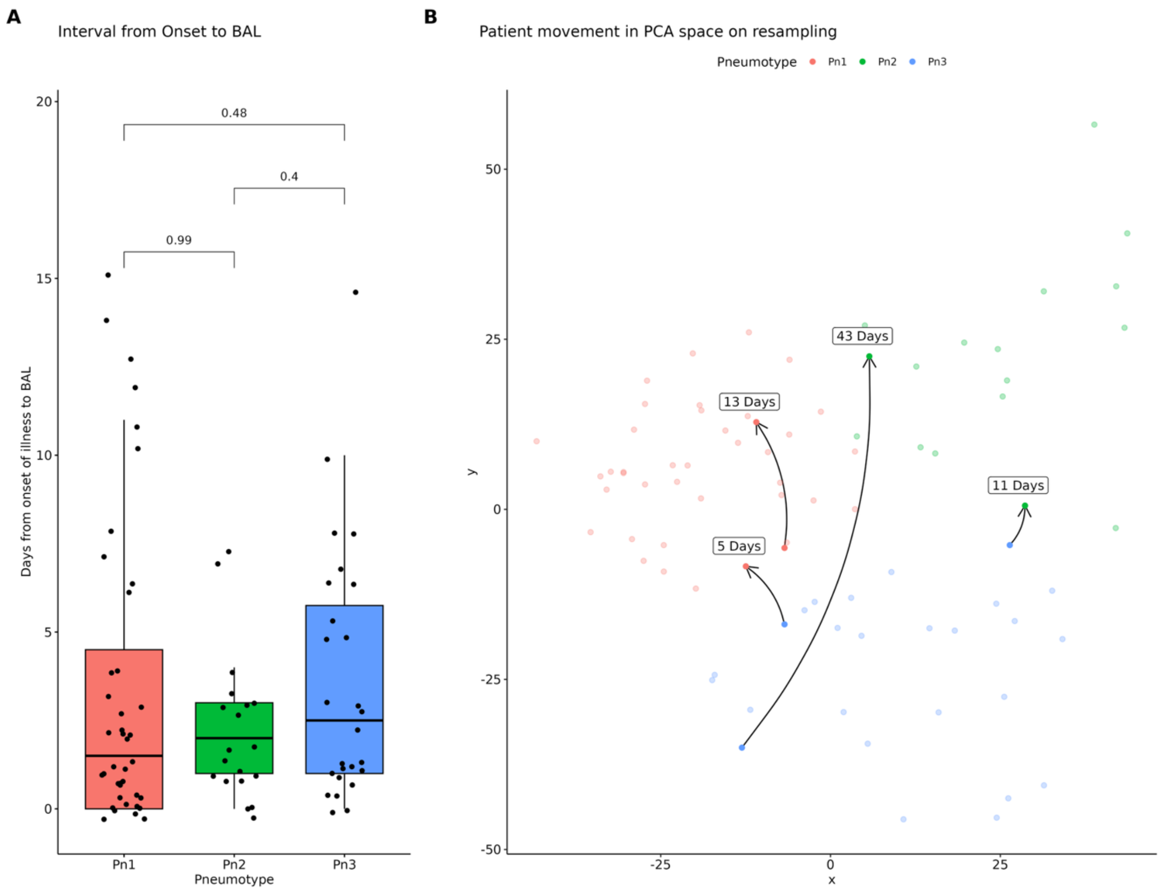

Supplementary Figure 4: Differential cell counts by Cytospin followed by Kwik-Diff™ staining with identification by morphology, subdivided into Pneumotype and normalized to lavage return volume.

A Bronchial epithelial cells. **B** Lymphocytes. **C** Macrophages. **D** Monocytes. **E** Neutrophils. **F** Other cells. **G** Red blood cells. **H** Total cell count. **I** Total cell count excluding red blood cells. Benjamini-Hochberg adjusted p-values: *=p<0.05, **=p<0.01, ***=p<0.001, ****=p<0.0001. N=39 Pn1, 19 Pn2 and 22 Pn3.

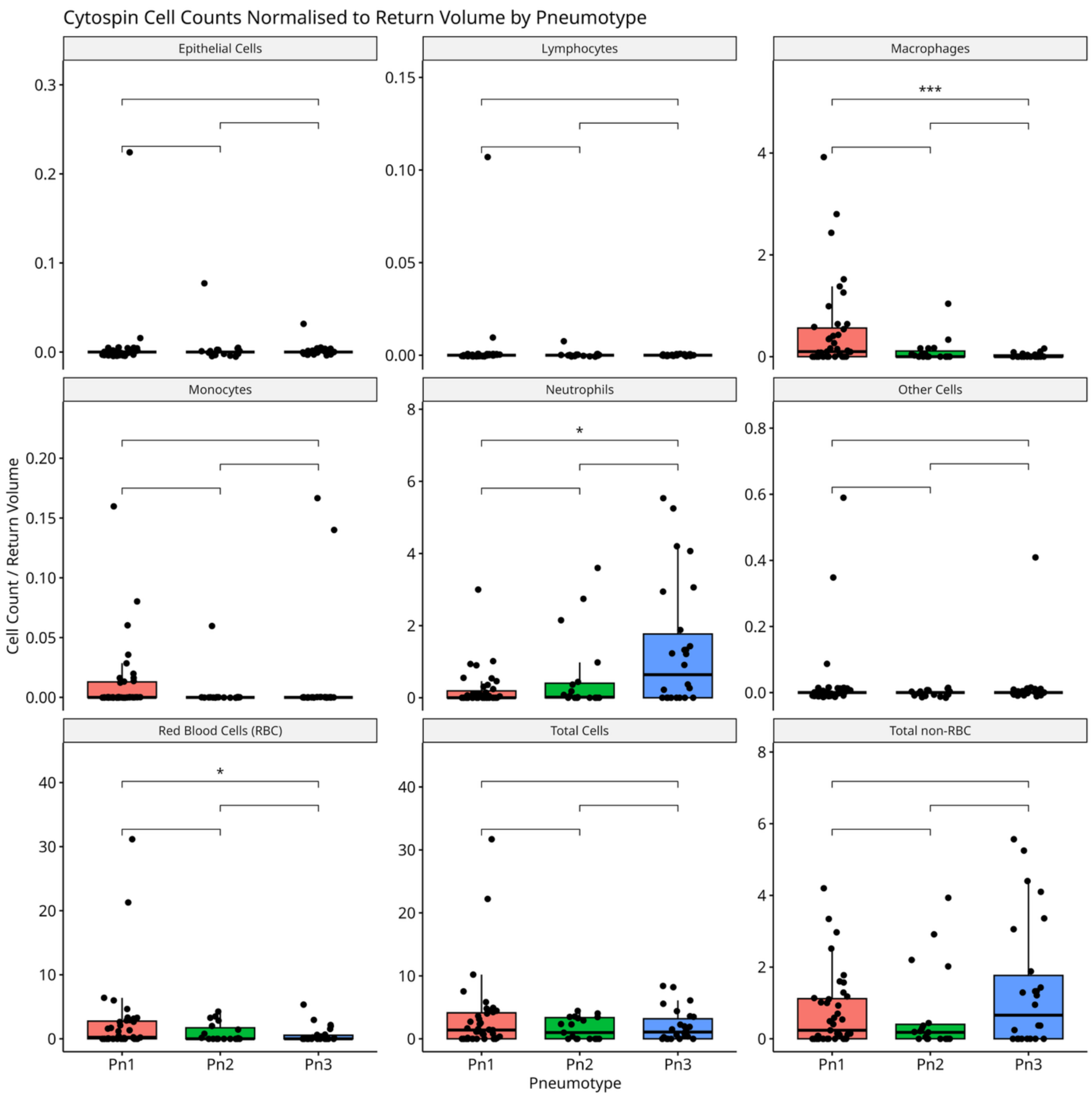

Supplementary Figure 5: Transcription Factor Enrichment Analysis by Pneumotype
Transcription Factor Enrichment Analysis using CHEA3[25] for up (left in yellow) and down (right in blue) regulated genes in each Pneumotype. The networks show known relationships between the top 10 enriched Transcription Factors. **A-B** Pneumotype 3, **C-D** Pneumotype 1, **E-F** Pneumotype 2. Edges between Transcription factors are defined by evidence from the ChEA3 libraries and are directed where ChIP-seq evidence supports the interaction.

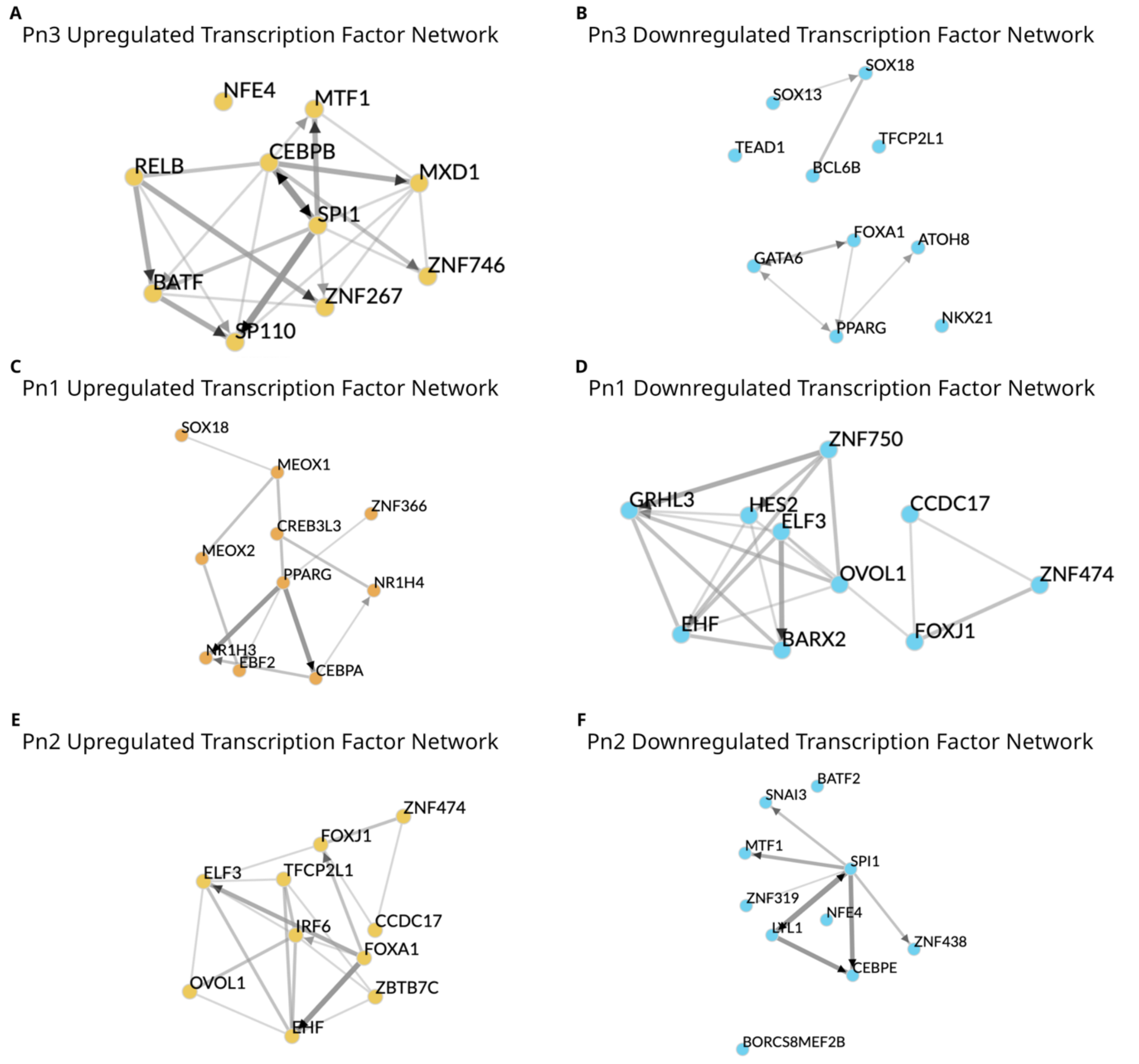

Supplementary Figure 6: Cytokine ratios and peripheral blood neutrophil counts by pneumotypes. A Selected pro- and anti-inflammatory cytokine ratios by pneumotypes. **B** Peripheral blood neutrophil counts by pneumotype. P-values by Kruskal-Wallis rank one way analysis of variance test (KW) and with Dunn's post-hoc pairwise test (non-significant pairwise values not shown for clarity).

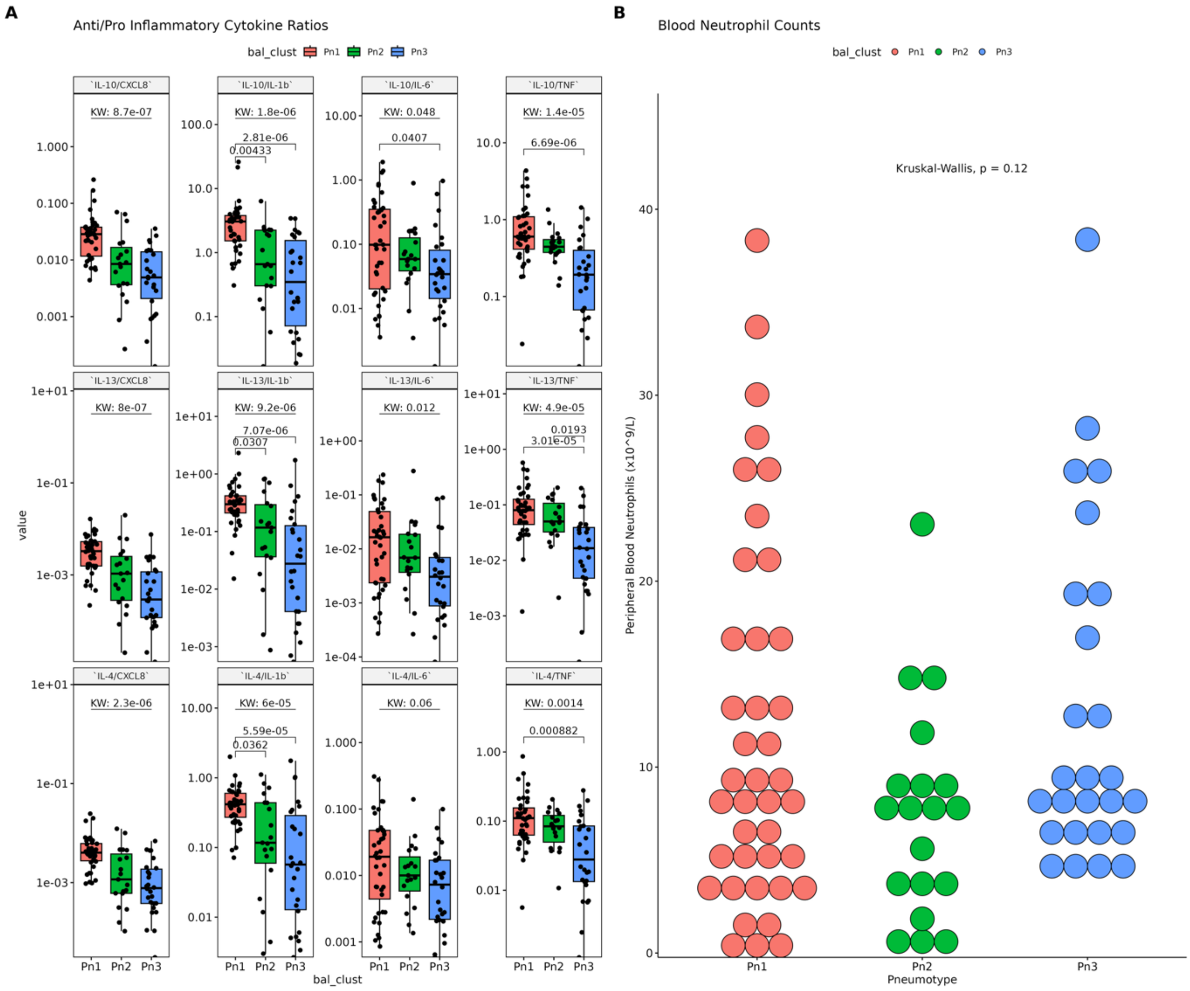

Supplementary Figure 7: BAL Weight Gene Co-Expression Network Modules. A Heatmap of module correlations with each Pneumotype, FDR-corrected Student asymptotic p values for correlation. N=39 Pn1, 19 Pn2 and 22 Pn3. **B-J** Over Representation Analysis of module hub genes for modules showing significant differences between Pneumotypes. Coloured by absolute -log10(p) to show differing magnitudes of significance between modules.

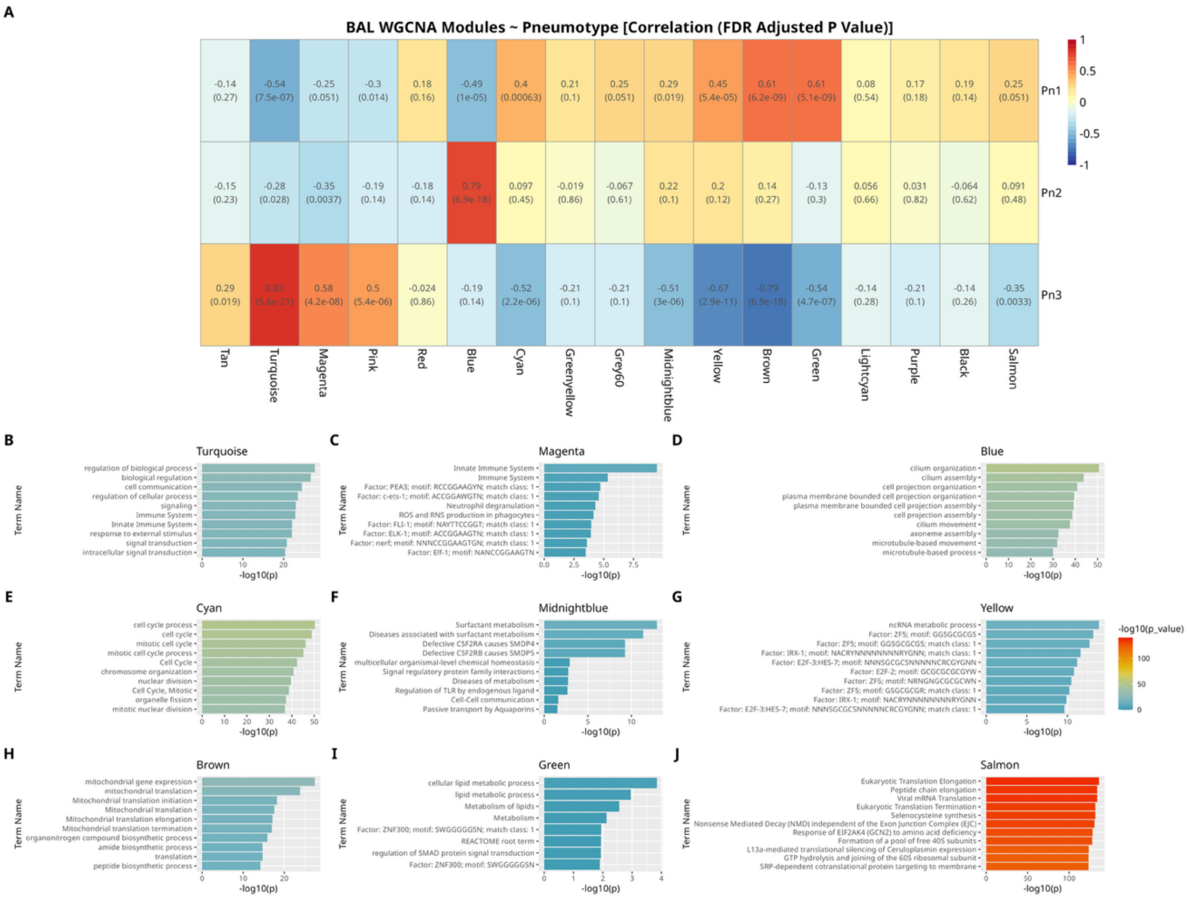

Supplementary Figure 8: Cellular composition for Langellier and Wauters validation cohorts

A Percentage of cells by major cell type from Wauters[48] scRNA annotation by W1-3 cluster. N=13 W1, 8 W2, 14 W3. P-values by Kruskal-Wallis (KW) with pairwise comparisons by Dunn's post hoc test (non-significant pairwise values not shown for clarity). **B** Principal components 1 and 2 of the 10% most highly variable genes from bronchoalveolar lavage pseudobulked single-cell RNA sequencing from Wauters et al [48] plotted with individuals coloured by W-cluster (W1-3). Arrows indicate Pearson's correlation between percentage of distal alveolar stem cells (DASCs) and neutrophil subsets from exploratory re-analysis of Wauters scRNA data and cluster. N=13 W1, 8 W2, 14 W3 **C** Estimated cellular proportions by xCell bulk RNA deconvolution of tracheal aspirate (TA) clusters TA1 (green) and TA2 (blue) from Langelier et al [21] p-values by two sided Wilcoxon rank sum N=35 TA1 and 49 for TA2. **D** Comparative estimated cellular proportions of TA from Langelier et al and BAL from bronchoalveolar lavage dataset reported in this paper, by xCell RNA deconvolution. P-values by two-sided Wilcoxon rank sum N=84 TA and 80 BAL. For all box and whisker plots show median (central line), IQR (box) and 1.5xIQR (whiskers) with individual data points shown as dots.

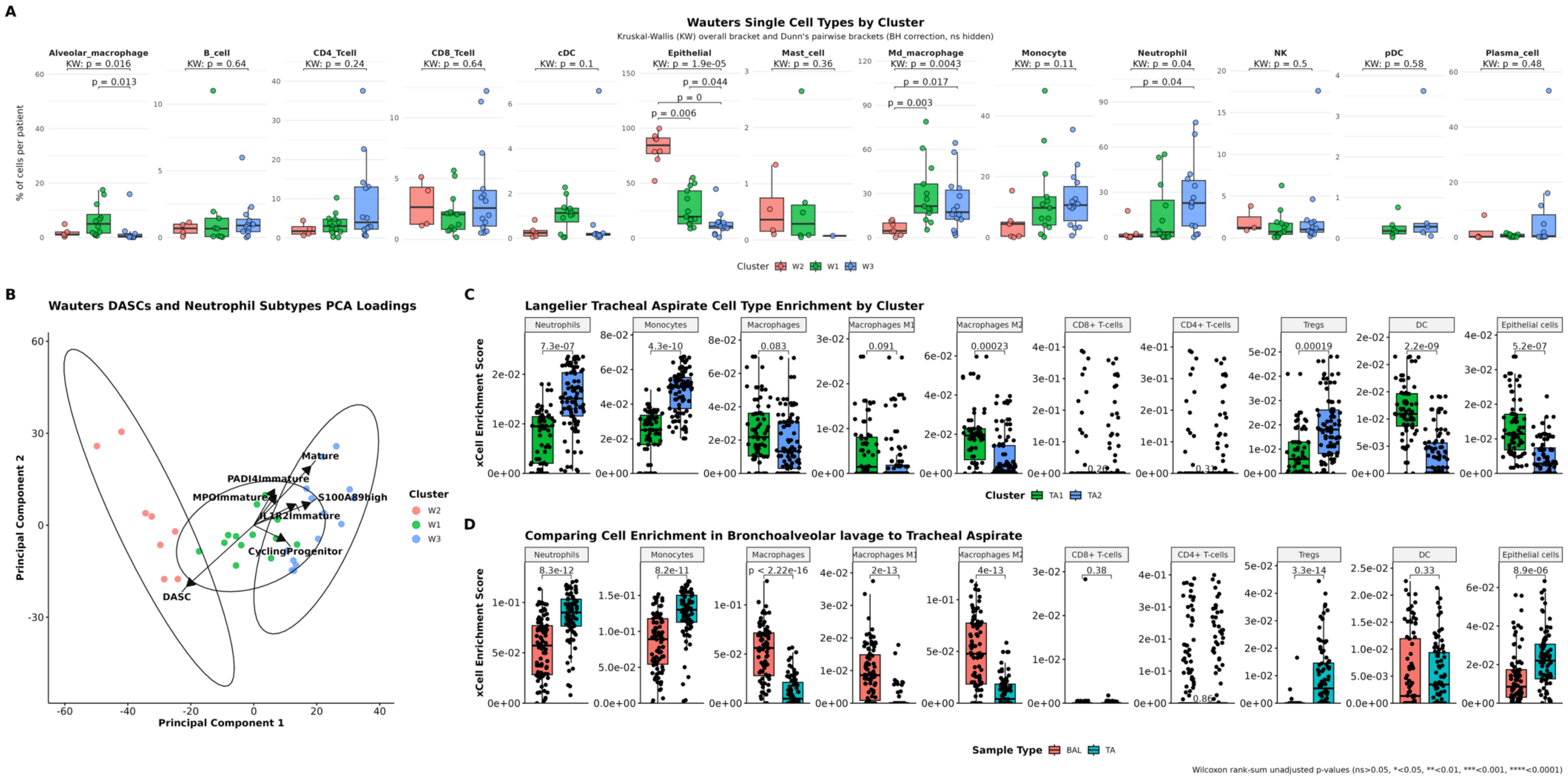
A
Wauters Single Cell Types by Cluster
Kruskal-Wallis (KW) overall bracket and Dunn's pairwise brackets (BH correction, ns hidden)
% of cells per patient
Alveolar_macrophage
KW: p = 0.016
p = 0.013
B_cell
KW: p = 0.64
CD4_Tcell
KW: p = 0.24
CD8_Tcell
KW: p = 0.64
cDC
KW: p = 0.1
Epithelial
KW: p = 1.9e-05
p = 0.044
p = 0
p = 0.006
Mast_cell
KW: p = 0.36
Md_macrophage
KW: p = 0.0043
p = 0.017
p = 0.003
Monocyte
KW: p = 0.11
Neutrophil
KW: p = 0.04
p = 0.04
NK
KW: p = 0.5
pDC
KW: p = 0.58
Plasma_cell
KW: p = 0.48
Cluster W2 W1 W3
B
Wauters DASCs and Neutrophil Subtypes PCA Loadings
Mature
PADI4Immature
MPOImmature
S100A89high
IL1R2Immature
CyclingProgenitor
DASC
Principal Component 1
Principal Component 2
Cluster
W2
W1
W3
C
Langelier Tracheal Aspirate Cell Type Enrichment by Cluster
xCell Enrichment Score
Neutrophils 7.3e-07
Monocytes 4.3e-10
Macrophages 0.083
Macrophages M1 0.091
Macrophages M2 0.00023
CD8+ T-cells 0.26
CD4+ T-cells 0.37
Tregs 0.00019
DC 2.2e-09
Epithelial cells 5.2e-07
Cluster TA1 TA2
D
Comparing Cell Enrichment in Bronchoalveolar lavage to Tracheal Aspirate
xCell Enrichment Score
Neutrophils 8.3e-12
Monocytes 8.2e-11
Macrophages p < 2.22e-16
Macrophages M1 2e-13
Macrophages M2 4e-13
CD8+ T-cells 0.38
CD4+ T-cells 0.86
Tregs 3.3e-14
DC 0.33
Epithelial cells 8.9e-06
Sample Type BAL TA
Wilcoxon rank-sum unadjusted p-values (ns>0.05, *<0.05, **<0.01, ***<0.001, ****<0.0001)

Supplementary Figure 9: Schematic representations of hypothesised mechanisms underpinning Pneumotypes. A Schematic representation of the self-reinforcing and mutually reinforcing cycles that form a bi-stable equilibrium that drives sustained inflammation in Pn3, allowing inflammation to persist after removal of the precipitating insult. **B** Schematic representation of the mutually exclusive natures of Pn1 and 3, whereby either tolerant tissue-resident macrophages dominate and exclude neutrophils (Pn1) or neutrophils polarise macrophages to an inflammatory phenotype and produce a persistent peripheral blood infiltrative phenotype (Pn3). Created with BioRender.com Conway Morris, A. (2026) https://BioRender.com/yu1jeu6 and https://BioRender.com/sye5wvo

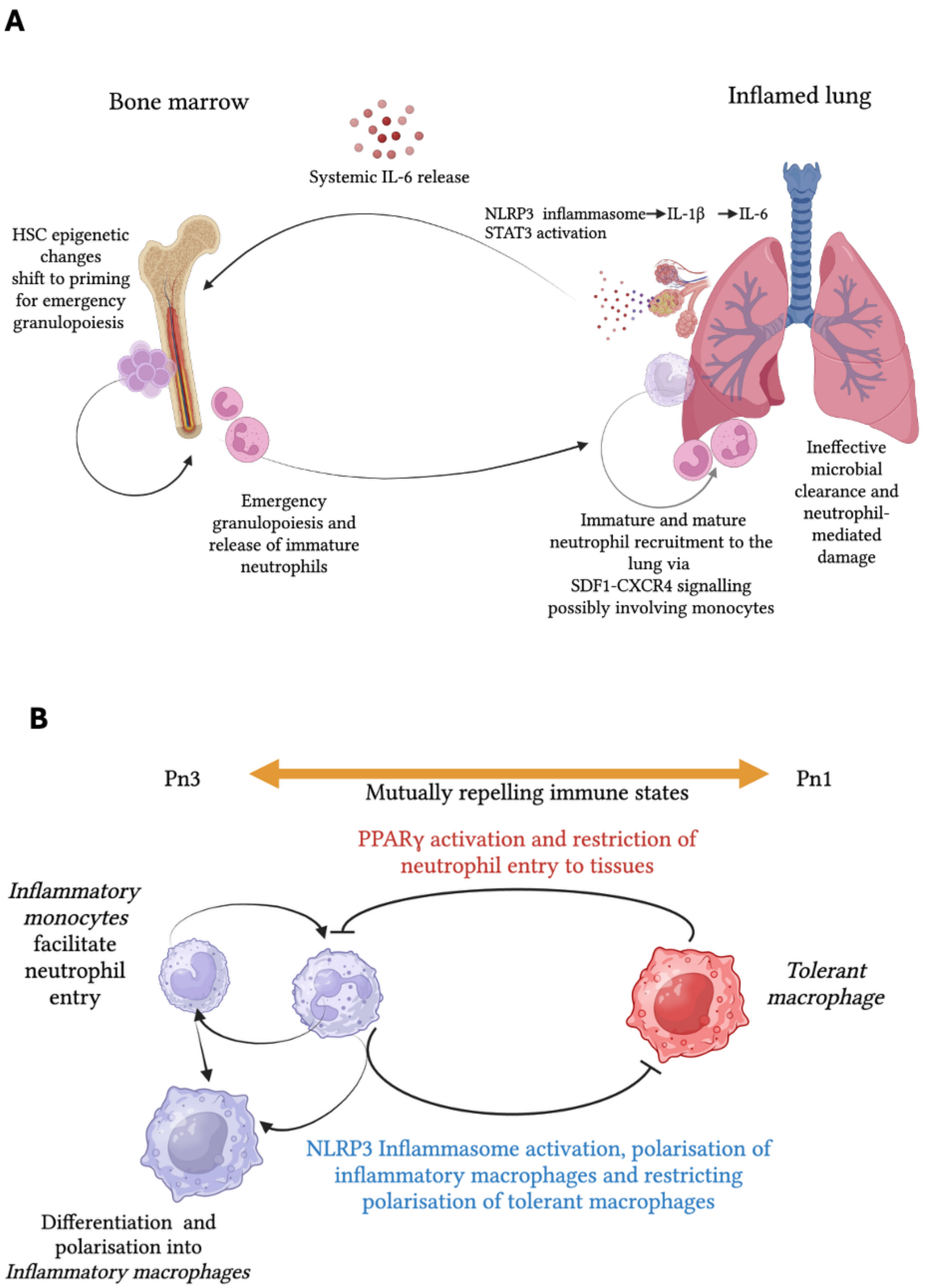
A
Bone marrow
Inflamed lung
Systemic IL-6 release
NLRP3 inflammasome→IL-1β →IL-6
STAT3 activation
HSC epigenetic changes shift to priming for emergency granulopoiesis
Emergency granulopoiesis and release of immature neutrophils
Immature and mature neutrophil recruitment to the lung via SDF1-CXCR4 signalling possibly involving monocytes
Ineffective microbial clearance and neutrophil-mediated damage
B
Pn3
Mutually repelling immune states
Pn1
PPARγ activation and restriction of neutrophil entry to tissues
Inflammatory monocytes facilitate neutrophil entry
Tolerant macrophage
NLRP3 Inflammasome activation, polarisation of inflammatory macrophages and restricting polarisation of tolerant macrophages
Differentiation and polarisation into Inflammatory macrophages
