# Supplementary material for: Pulmonary inflammation in severe pneumonia is characterised by compartmentalised and mechanistically distinct sub-phenotypes
Source: Nat Commun. 2026 Jun 23;17:5312. doi: 10.1038/s41467-026-74190-x (PMC13291267; doi:10.1038/s41467-026-74190-x)
Supplement: Supplementary file 2 — Description of Additional Supplementary Files [file 41467_2026_74190_MOESM2_ESM.pdf]

Description of Additional Supplementary Files

Supplementary Data 1: Contains abbreviated clinical meta-data, pathogen PCR from Taqman array card, BAL and serum cytokines, cytology cell counts, BAL gene counts and Blood gene counts
