# Supplementary material for: Pulmonary inflammation in severe pneumonia is characterised by compartmentalised and mechanistically distinct sub-phenotypes
Source: Nat Commun. 2026 Jun 23;17:5312. doi: 10.1038/s41467-026-74190-x (PMC13291267; doi:10.1038/s41467-026-74190-x)
Supplement: Supplementary file 4 — Transparent Peer Review file [file 41467_2026_74190_MOESM4_ESM.pdf]

nature portfolio

Peer Review File

Pulmonary inflammation in severe pneumonia is characterised by compartmentalised and mechanistically distinct sub-phenotypes

Corresponding Author: Dr Andrew Conway Morris

This file contains all reviewer reports in order by version, followed by all author rebuttals in order by version.

Version 0:

Reviewer comments:

Reviewer #2

(Remarks to the Author)
This study by Heffrey at al. presents a comprehensive analysis off 80 patients with clinically suspected severe pneumonia using bulk RNAseq of BAL cells alongside clinical data. The authors identify three clusters termed "Pneumotypes" based on bulk RNAseq of BAL cells from these patients, which are then further characterized by BAL cytokine profiles, serum cytokines, and RNAseq of blood cells (where limited correlations were found). The Pneumotypes show trends in survival and significant differences in extubation probability, but these differences between the clusters appear to be mainly driven by cell composition. This raises concerns about the clinical relevance of defining Pneumotypes by bulk RNAseq (see Major Comment 1). While the datasets provided in this study represent a potentially valuable resource (and some evidence for similar clusters is found in other publications using tracheal aspirates or bulk RNAseq of alveolar macrophages, repectively), direct validation of these subtypes is lacking, which is critical to substantiate the findings (see Major Comment 2). The technical methods and bioinformatics approaches are sound and thoroughly done, but several of the conclusions drawn are not fully supported by the data presented in the paper and should be revised (see Major Comment 3). Additionally, the presentation of data should be seriously improved for clarity (see Major Comment 4&5).

Major Comments:
1) Relevance of Pneumotypes: The differential expression observed in BAL fluid (BALF) seems to be largely influenced by cell composition (e.g., Pneumotype 3 - increased neutrophil counts, upregulated DEGs are associated with neutrophils). In a clinical setting, what is the benefit of a RNAseq analysis compared to conventional cytological methods such as flowcytometry of BAL? In addition, neutrophils are known to degrade quickly and release RNases, which can degrade RNA, raising concerns about the accuracy of downregulated genes in Pneumotype 3. It would be beneficial to include a plot showing the RNAseq library in the Supplements as well as the PCA plot (Figure 1) colored by library size, to ensure that the clustering is not driven by technical artefacts, such as RNA degradation or low cell numbers (as seen with Pneumotype 2 which has fewer cells compared to the other groups).
2) Validation of Pneumotypes: Although the authors note the absence of directly comparable public datasets and do find evidence of similar clusters in other studies (one using tracheal aspirates, the other bulk RNAseq of alveolar macrophages), the paper would benefit greatly from further validation. The authors could attempt to validate these Pneumotypes using pseudobulk analysis of single-cell RNAseq datasets more directly comparable to this study (e.g. Wauters et al., https://doi.org/10.1038/s41422-020-00455-9).
3) General conclusions: Several deductions are drawn based solely on RNAseq data and are not supported by cytospin data. For instance, monocyte signatures are enriched in Pn3 based on deconvolution (Fig. 1H), but this finding is not corroborated by cytospin data (Extended Data Fig. 3D), which shows monocyte levels are not elevated. This discrepancy should be acknowledged and discussed in the manuscript. As such, the statement "monocytes are also expanded in Pn3" (Line 255) should be revised. Similarly, in the discussion, the authors speculate that the "lung monocyte-neutrophil co-recruitment in Pn3…may form a positive feedback loop" (Line 481-483). However, given that cytospin data for this Pneumotype show minimal to no monocytes (Extended Data Fig. 3), drawing such conclusions based on an enrichment score, without corroborating evidence from actual cell counts, seems unwarranted.
Similarly, all other deductions made based on the deconvolution but not confirmed by other methods should be phrased more carefully (E.g., Line 248: "Alveolar neutrophilia was found in both deconvolution and cytology…"). While the deconvolutions shows an enrichment of neutrophil signatures and the cytospin in Fig. 3E shows increased neutrophils, it

looks like nearly half of the samples do not have elevated neutrophil cell counts. The authors should therefore do a statistical testing to see if this elevation is significant compared to the other Pneumotypes.
4) Readability
- Gene names should be in italics and proteins non-italicised (see Guidelines for Human Gene Nomenclature, https://doi.org/10.1038/s41588-020-0669-3).
- It would drastically increase the readability and also impact of the paper if findings mentioned in the text would be underlined by a figure. In multiple cases, the authors refer to the Supplemental Results file sheet, where it is hard to find the corresponding data and in my opinion, should also not be expected from the reader of the paper. At the same time, significant data shown in the main figures are not even mentioned in the text.
E.g.
Line 214: The authors note a significant difference of plasma IL-1ra between Pneumotypes, but a reference is only made to the supplemental data sheet and it is also not clear, between which Pneumotypes this difference arises.
Line 251-252: The enrichment of G-CSF (transcript, BALF protein level, plasma level) is not shown in the paper.
Line 255-256: “monokines CCL3 and CCL4 at both transcript and protein level” – the protein level should be shown in a figure.
Line 259: “elevation of …IL-10” – cytokine levels should be shown in a figure.
Line 340-342: “…indicated innate immune activity, which is consistent with the moderate levels of infiltrating immune cells identified by deconvolution and cytology” – In Extended Figure 3E it seems that Pn2 has the least number of cells in every cluster, how do you explain the enrichment of innate immune activity compared with the other clusters?
Line 356-357: “…only 100 genes in Pn3 (Supplemental Results Sheet 9) which mapped to neutrophil degranulation…” this result should be shown in a figure since it also underlines the neutrophil signatures in Pn3.
5) Figures
- The figures are generally far too small and the font sizes & plots must be enlarged. In addition, the relevance and information provided in several network plots (which are generally not readable) is questionable.

- Similarly to major Comment 4, findings shown in the figure should be mentioned and explained in the text;
Figure 1E: It is mentioned in the text and in Extended Data Figure 2B that 4 cases were sampled twice, but why is this not also visible in Figure 1E?
Figure 1I: CXCL8 and Il-1b increase in Pn3 should be mentioned in the text.
Figure captions (e.g. TF enrichment (up/down) in Figure 2C/D, 3C/D, 4C/D) would help the reader to understand the paper better.
Figure 5 & 6C: The X labels/Y labels showing the gene modules e.g. “BMEpurple” are making the figure not very informative and should be renamed to corresponding names of enrichment patterns (e.g. “Cell cycle processes”).
Figure 6D: Although there are different Pneumonia Types shown in the legend, mentioned in the text and used by the study from Grant et al., the figure only shows COVID-19 Pneumonia types. Is this really correct? If yes, the authors should clarify this in the text and comment on how this fits with their study, where e.g. Pn3 clearly mainly bacterial pneumonia cases.
Extended Data Figure 3 – Could the numbers also be normalized to the volume of BALF? In this regard, it might give additional information to show percentages of cells.

Minor comments:
According to the methods, the authors sequenced the BALF cells after spinning them down, but in the text it is written multiple times “sequencable lavage”. For clarification, this wording should be rephrased to “sequencable lavage cells” or “BALF cells”.
Since plasma cytokines (which were sampled in this study, see Methods Line 897) are not the same as serum cytokines, it should be written plasma cytokines throughout in the study.

Reviewer #3

(Remarks to the Author)
The manuscript by Jeffrey, M. et al., examined transcriptome, cytokines, and microbiology using bronchoalveolar lavage fluids and blood collected from patients admitted to intensive care units and placed on mechanical ventilation for severe pneumonia. They identified three distinct transcriptional changes which they termed Pneumotype (Pn) 1, 2, and 3. All three Pns had comparable clinical presentations but showed divergent outcomes. Overall these studies are interesting and provide new insights into patients’ immune-based responses to microbial infections of the respiratory tract. Below are several points that need clarification:

1) Figure 1A. A clearer description of elements used to separate each Pn subtype should be provided to clarify the rationale for the distinct phenotypes because all other comparisons rely on the original observation.
2) Figure 1G. Within the first one post-extubation, nearly ¼ of all Pn types die, and one-year survival ranges from 50 to 75%. This poor overall outcome is not consistent with the rate of survival in the 40-70 year old age groups. Are there any specific factors in this cohort that separate them from other studies?
3) Figure 1I. It is unclear why the authors chose to compare some of the groups. For example, BAL and serum concentrations are randomly compared among Pn1, Pn2, and Pn 3 groups.
4) Figure 2A. Their analysis based on data shown in Fig 1 points to Pn2 as more distinct than Pn1 and Pn3. Therefore, the rationale for comparing the Pn3 group to the combined two groups is unclear.
5) Figure 2E. The large network of interactome could be reduced to increase clarity.
6) Extended data Figure 2B. Three out of 4 repeat BAL sample analysis shows they changed the Pn category from Pn3 to Pn 2 or Pn1. Can the authors speculate how this dynamic change in Pn endotype could be interpreted in the overall scheme

of their cohort analysis?
7) Figure 3. Here, it is unclear why Pn1 is compared to the combined Pn2 and Pn3 since the latter two subtypes show significantly different outcomes.
8) Figure 3G. This diagram could be reduced to improve clarity.
9) Figure 4. This is perhaps the most important analysis because here the authors compare the cohort that has different outcomes from Pn1, and Pn3 with similar clinical outcomes. However, much like the other gene/protein interactome data in Fig 2E and Fig. 3G, the diffuse data presentation is difficult to comprehend.
10) Figure 5. The main information embedded within this figure is difficult to discern. Do the authors believe that blood-based genomics and proteomics do not distinguish Pn1, Pn2, and Pn3? Is a higher or lower confidence level attributable to BAL vs blood biomarkers?

Reviewer #4

(Remarks to the Author)
I co-reviewed this manuscript with one of the reviewers who provided the listed reports. This is part of the Nature Communications initiative to facilitate training in peer review and to provide appropriate recognition for Early Career Researchers who co-review manuscripts.

Version 1:

Reviewer comments:

Reviewer #2

(Remarks to the Author)
While several of our concerns have been satisfactorily addressed by the authors and the overall readability of the paper has improved, one major concern remains. The validation of Pneumotypes in an independent dataset would considerably strengthen the findings and potentially confirm the importance for future use (See A2). Further, although improved, Figure design and flow of results require serious attention. All labels in this response correspond to the point-by-point reply.

Concerns:
A2) Validation of Pneumotypes
The authors` efforts to identify suitable RNA-seq datasets are commendable, and it is unfortunate that no appropriate datasets were made available. However, we do not agree with the authors` argument that an additional COVID-19 dataset is useless, because their comparison with the Grant et al. dataset (of COVID-19 patients) display a distinct phenotype. In our opinion, Grant et al. data are RNA-seq of macrophages from COVID-19 patients, which is technically different from bulk RNA-seq from BALF (this study). We therefore repeat our earlier suggestion to validate the data using pseudobulk analysis of the suggested single-cell RNA-seq dataset (Wauters et al.), which includes 22 COVID-19 and 13 non-COVID-19 pneumonia patients (Wauters et al., https://doi.org/10.1038/s41422-020-00455-9). The authors have neither attempted to analyze nor addressed this suggestion in the point-by-point response, and we believe it could substantially strengthen this study.

A3+4) Figures and readability
We appreciate that the authors have added statistical tests, the proposed clarifications, and revised most figures to improve readability. These changes are clear improvements. Nevertheless, several figure panels remain difficult to interpret due to their small size and font. It may be helpful to print out the figures to assess which elements are easily readable and which are not. Another approach could be to ask colleagues or non-experts to look at the figures and indicate whether they can quickly grasp the main message and details. Simplifying or enlarging certain panels and prioritizing selected results to display in the main figures could greatly enhance clarity. Ultimately, figures are most effective when they can be understood at a glance without overwhelming the reader.

In addition: while the results section has improved overall, the narrative still jumps back and forth between figures when the findings are explained. A more linear organization would likely make the text much easier to follow.
Also, the figure legends have been revised and are more informative, but we suggest focusing them on describing the methods and figure-content, while leaving interpretation to the main text.

A5) WGCNA module analysis
The authors` rationale for using this analysis to compare the different BALF, blood and SRS clusters on a larger scale is understandable. However, Figure 5 only presents blood RNA clusters of the 3 Pneumotypes (while the BALF RNA clusters are compared in Extended Data Figure 6), which should be clearly indicated in the figure description (it is now written “lung and blood cluster correlations” - which is confusing).

A12)
We find the normalized version of the BALF cell counts to be more informative and precise and would recommend replacing the original version. Additionally, it would be more meaningful to label the actual cell concentration (e.g. Cell Count/mL) instead of Cell Count/ Return volume.

Reviewer #3

(Remarks to the Author)
The manuscript has improved in data presentation and concept. I have no further concerns.

Reviewer #4

(Remarks to the Author)
I co-reviewed this manuscript with one of the reviewers who provided the listed reports. This is part of the Nature Communications initiative to facilitate training in peer review and to provide appropriate recognition for Early Career Researchers who co-review manuscripts.

Version 2:

Reviewer comments:

Reviewer #2

(Remarks to the Author)
the authors have addressed our concerns, we have no further comments.

Reviewer #4

(Remarks to the Author)
I co-reviewed this manuscript with one of the reviewers who provided the listed reports. This is part of the Nature Communications initiative to facilitate training in peer review and to provide appropriate recognition for Early Career Researchers who co-review manuscripts.

Open Access This Peer Review File is licensed under a Creative Commons Attribution 4.0 International License, which permits use, sharing, adaptation, distribution and reproduction in any medium or format, as long as you give appropriate credit to the original author(s) and the source, provide a link to the Creative Commons license, and indicate if changes were made.
In cases where reviewers are anonymous, credit should be given to 'Anonymous Referee' and the source.
The images or other third party material in this Peer Review File are included in the article's Creative Commons license, unless indicated otherwise in a credit line to the material. If material is not included in the article's Creative Commons license and your intended use is not permitted by statutory regulation or exceeds the permitted use, you will need to obtain permission directly from the copyright holder.
To view a copy of this license, visit https://creativecommons.org/licenses/by/4.0/

Response to reviewers comments for **Pulmonary inflammation in severe pneumonia is characterised by compartmentalised and mechanistically distinct sub-phenotypes.**

Reviewer #2 (Remarks to the Author):
This study by Heffrey at al. presents a comprehensive analysis off 80 patients with clinically suspected severe
pneumonia using bulk RNAseq of BAL cells alongside clinical data. The authors identify three clusters termed
“Pneumotypes” based on bulk RNAseq of BAL cells from these patients, which are then further characterized by BAL cytokine profiles, serum cytokines, and RNAseq of blood cells (where limited correlations were found). ThePneumotypes show trends in survival and significant differences in extubation probability, but these differences between the clusters appear to be mainly driven by cell composition. This raises concerns about the clinical relevanceof defining Pneumotypes by bulk RNAseq (see Major Comment 1). While the datasets provided in this study representa potentially valuable resource (and some evidence for similar clusters is found in other publications using trachealaspirates or bulk RNAseq of alveolar macrophages, repectively), direct validation of these subtypes is lacking, whichis critical to substantiate the findings (see Major Comment 2). The technical methods and bioinformatics approachesare sound and thoroughly done, but several of the conclusions drawn are not fully supported by the data presented inthe paper and should be revised (see Major Comment 3). Additionally, the presentation of data should be seriouslyimproved for clarity (see Major Comment 4&5).

Major Comments:
R1) Relevance of Pneumotypes: The differential expression observed in BAL fluid (BALF) seems to be largely
influenced by cell composition (e.g., Pneumotype 3 - increased neutrophil counts, upregulated DEGs are associated with neutrophils). In a clinical setting, what is the benefit of a RNAseq analysis compared to conventional cytological methods such as flowcytometry of BAL? In addition, neutrophils are known to degrade quickly and release RNases,which can degrade RNA, raising concerns about the accuracy of downregulated genes in Pneumotype 3. It would bebeneficial to include a plot showing the RNAseq library in the Supplements as well as the PCA plot (Figure 1) colored by library size, to ensure that the clustering is not driven by technical artefacts, such as RNA degradation or low cell numbers (as seen with Pneumotype 2 which has fewer cells compared to the other groups).

A1) We agree with the reviewer that a significant component on the pneumotypes does appear to be driven by different cellular make-up. This is supported by the correspondence between the BAL cellular enrichment by RNA deconvolution presented in revised Figure 2A and the cytology in Extended data Figure 4. Importantly, however, this is not the only driving factor in cluster assignment. Firstly, cytology alone cannot differentiate between different cell subsets, most notably the macrophage phenotypes that distinguish Pn1 from Pn3, and the neutrophil maturity signatures in Pn3. In addition, as the reviewer notes in their next comment, whilst cytology and RNA deconvolution show similar patterns there is not a one-to-one correspondence. Second, some signatures, most notably those for epithelial cell and epithelial cell repair in Pn2, are in stark excess of the number of identified cells by cytology leading us to speculate as to the nature of cells that are driving this signature

(discussion line 538-542, distal alveolar stem cells). Finally, we note that previous investigations of bronchoalveolar lavage cellularity by ourselves and others (references 8 and 11 in the manuscript) had not identified these clusters, and it was only by analysing the BAL transcriptome that we were able to identify them and ascribe the mechanistic features described. This is not to say that simpler methods of pneumotype identification will not be possible in the future, indeed in the original manuscript we used flow cytometric data from Grant and colleagues to identify Pn1 and 3 in an external dataset. This would, however, require further validation with simultaneous determination of Pneumotype by RNA profile (as our "gold standard") and then comparison of cell-surface markers by techniques such as flow cytometry to establish their diagnostic performance. Indeed, this would be one potential route to developing a clinically applicable test that would allow stratification of patients for subsequent clinical trials.

We have modified the discussion to note the role of differential cellularity, the potential for techniques other than RNAseq to be used to identify pneumotypes and the need to validate these approaches in future work (lines 547-551 now read "Although we used RNA sequencing to identify the pneumotypes, translating this into a clinically useable test may require different approaches. The differential cellular makeup and cellular phenotypes may be assessable using tools such as flow cytometry [45] or parsimonius gene expression signatures, however these will require validation in external datasets with simultaneous capture of these data types."*).*

Regarding the issue of neutrophil-mediated RNA degradation, the reviewer makes a valid point. We have undertaken the suggested analysis of library size by pneumotype (now included in the Extended Data Figure 2). We have added a sentence to note this finding in the initial results section. Line 204-208 now reads "To examine whether the cellular composition of the pneumotypes may have altered RNA content, for instance through RNA degradation by neutrophils, we compared total counts and numbers of unique genes sequenced. This did not identify any systematic differences between the pneumotypes (Extended Data Figure 2)."

R2) Validation of Pneumotypes: Although the authors note the absence of directly comparable public datasets and do find evidence of similar clusters in other studies (one using tracheal aspirates, the other bulk RNAseq of alveolar macrophages), the paper would benefit greatly from further validation. The authors could attempt to validate these Pneumotypes using pseudobulk analysis of single-cell RNAseq datasets more directly comparable to this study (e.g. Wauters et al., https://doi.org/10.1038/s41422-020-00455-9).

A2) *We agree with the reviewer that further external validation would be valuable, as we note in the discussion (lines 527-530). We have conducted a systematic review of the literature (using search terms "(pneumonia) and (bronchoalveolar lavage) and (RNA) limited to 'humans')" which identified 449 titles in pubmed. We also examined Gene expression omnibus (GEO) datasets using "(pneumonia)AND(bronchoalveolar lavage)AND(Homo sapiens)" which identified 88 records. Those studies which report RNA sequencing data from patients with pneumonia are unfortunately not suitable for validation. The majority of identified studies concerned patients with COVID-19 who, as we demonstrate in the Grant dataset, have a distinct pulmonary phenotype. Whilst some include bacterial pneumonia cases these make a very small number (typically 2-5 patients) which could not be meaningfully clustered.*

We did identify a single study which could possibly have been suitable (Xiao K, Cao Y, Han Z, et al. A pan-immune panorama of bacterial pneumonia revealed by a large-scale single-cell transcriptome atlas. Signal Transduct Target Ther. 2025 Jan 6;10(1):5). This study reported single-cell RNAsequencing from 36 patients with severe pneumonia. Unfortunately, despite having indicated that the RNAsequencing data had been publicly deposited in line with journal requirements in the Chinese National Centre for Bioinformatics database these data are not accessible. We have written to the investigators on several occasions requesting either public release of the data, access to the data and have offered to send code to allow analysis to be done at their site, however this has not been met with any response. We also tried reaching out through intermediary contacts within the Chinese National Centre, but again were unable to even secure a reply let alone access to the data. This is obviously disappointing, but we do not envisage this data being made public, and the data contained in the manuscript and supplemental sections does not include individual patient level data to enable even partial replication.

We have further reached out to contacts within the Intensive Care and Respiratory Infection community to seek to identify unpublished datasets that could be used for validation. Unfortunately, none of the investigators we contacted were able to supply bronchoalveolar lavage data from either bulk or single cell RNA sequencing, and all indicated the existing Langellier and Grant datasets as suggested ones to use, as we already have. Regrettably we therefore are unable to supply validation beyond what is already in the paper at this time. We do agree that further validation is required before we can use these phenotypes for clinical studies, as we indicate in the manuscript and are currently applying for funding to undertake this work. However we believe that the existing validation in both Grant and Langelier datasets replicates each Pneumotype externally and cross validates our RNA deconvolution results with flow-cytometry.

R3) General conclusions: Several deductions are drawn based solely on RNAseq data and are not supported by cytospin data. For instance, monocyte signatures are enriched in Pn3 based on deconvolution (Fig. 1H), but this finding is not corroborated by cytospin data (Extended Data Fig. 3D), which shows monocyte levels are not elevated. This discrepancy should be acknowledged and discussed in the manuscript. As such, the statement “monocytes are also expanded in Pn3” (Line 255) should be revised. Similarly, in the discussion, the authors speculate that the “lung monocyte-neutrophil co-recruitment in Pn3…may form a positive feedback loop” (Line 481-483). However, given that cytospin data for this Pneumotype show minimal to no monocytes (Extended Data Fig. 3), drawing such conclusions based on an enrichment score, without corroborating evidence from actual cell counts, seems unwarranted. Similarly, all other deductions made based on the deconvolution but not confirmed by other methods should be phrased more carefully (E.g., Line 248: “Alveolar neutrophilia was found in both deconvolution and cytology…”). While the deconvolutions shows an enrichment of neutrophil signatures and the cytospin in Fig. 3E shows increased neutrophils, it looks like nearly half of the samples do not have elevated neutrophil cell counts. The authors should therefore do a statistical testing to see if this elevation is significant compared to the other Pneumotypes.

A3) We agree with the reviewer that whilst RNA deconvolution and cytology were broadly in accordance for some cells the enrichment of monocytes in Pn3 identified by deconvolution was not replicated in the cytology. Furthermore, the correspondence between cytology and

RNA deconvolution was not perfect. The reasons for this are multiple but include cytospins reflecting a very small proportion of total cellularity. Furthermore, deconvolution indicates the relative proportions of a given cell type between conditions rather than absolute cell numbers. Therefore, it is possible that a relatively small population of monocytes may show differential abundance between the pneumotypes but not be apparent on qualitative but not fully quantitative cytopsins. Of note, in the validation undertaken in the Grant cohort (Figure 6D) we did identify expansion in monocytes in Grant 3 cluster (analogous to Pn3) as we indicated in lines 426-427.

We have acknowledged this discrepancy in the revised manuscript in several places. Line 257 in the results now acknowledges that the enrichment of monocytes was only seen at the transcript level reading "and monocytes are also transcriptionally enriched in Pn3"
In the revised discussion we now note in line 473 that "The neutrophil recruitment in Pn3, possibly sustained by monocyte co-recruitment..."
Whilst in the limitations section, lines 542-547, we now note "Similarly, although monocyte enrichment in Pn3 was noted by deconvolution of RNA transcripts, and in the validation data set from Grant et al [45] we did not identify a similar pattern by cytology from the derivation cohort lavage. Cytology, sampling a very small proportion of the total cell population, is less sensitive than RNA deconvolution and more granular phenotyping of immune cells will be required to confirm or refute this finding."

Regarding the comments on the neutrophils, we have added the requested statistical testing to the cytology data (Extended figure 4) which supports our previously statements regarding differential enrichment for macrophages (expanded in Pn1), neutrophils (expanded in Pn3) and red blood cells (expanded in Pn1). All other cell types showed no significant differences.

R4) Readability
We have made the changes suggested by the reviewer, to address each sub-comment in detail:

R4.1 Gene names should be in italics and proteins non-italicised (see Guidelines for Human Gene Nomenclature,
https://doi.org/10.1038/s41588-020-0669-3).
A4.1 These have now been corrected, where we refer to enrichment of a gene and its corresponding protein we have left these non-italicised for clarity. Transcription factors have been left non-italicised when inferred from the transcripts produced, but if detected at gene transcript level have been italicised.

R4.2 It would drastically increase the readability and also impact of the paper if findings mentioned in the text would be underlined by a figure. In multiple cases, the authors refer to the Supplemental Results file sheet, where it is hard to find the corresponding data and in my opinion, should also not be expected from the reader of the paper. At the same time, significant data shown in the main figures are not even mentioned in the text.
E.g.
Line 214: The authors note a significant difference of plasma IL-1ra between Pneumotypes, but a reference is only made to the supplemental data sheet and it is also not clear, between which Pneumotypes this difference arises.
Line 251-252: The enrichment of G-CSF (transcript, BALF protein level, plasma level) is not shown in the paper.
Line 255-256: "monokines CCL3 and CCL4 at both transcript and protein level" – the protein level should be shown in a figure.

Line 259: "elevation of …IL-10" – cytokine levels should be shown in a figure.

A4.2 We agree with the reviewer that these data should be presented in the main paper and not relegated to a supplemental datasheet only. In light of the reviewer's comment and other comments we have heavily revised the figures to improve clarity, reducing the amount of data in some figures and using the space to add the cytokines discussed. Revised figure 2 contains all the cytokines indicated above whilst Extended Data Tables 1 and 2 include the results for all measured proteins.

R4.3 Line 340-342: "…indicated innate immune activity, which is consistent with the moderate levels of infiltrating immune cells identified by deconvolution and cytology" – In Extended Figure 3E it seems that Pn2 has the least number of cells in every cluster, how do you explain the enrichment of innate immune activity compared with the other clusters?

A4.3 We note the reviewers comment referring to the cytology and deconvolution, and agree that immune cell infiltration is not a notable feature of Pn2. Where there is a clearer picture of moderate innate immune activity is in lavage inflammatory proteins (Figure 2B) and differential gene expression (Figure 4 C and D). We have therefore adjusted line 340-342 to read "…..we identified intermediate levels of lavage cytokines and inflammatory proteins (Figure 2B), with gene expression enriched for cytokine signalling and innate immune function (Figure 4C and D)"

R4.5) Line 356-357: "…only 100 genes in Pn3 (Supplemental Results Sheet 9) which mapped to neutrophil degranulation…" this result should be shown in a figure since it also underlines the neutrophil signatures in Pn3.

*A4.5 We have added an annotated volcano plot to Figure 5 (panel Figure 5B) to illustrate the gene expression in the blood of Pn3 vs non-Pn3. We indicate in the text (lines 355-359) that "….*and only 100 genes in Pn3 (Figure 5B) which mapped to neutrophil de-granulation [p=1.043e-15] and innate immune system [p=6.346e-8] Reactome terms"

R5) The figures are generally far too small and the font sizes & plots must be enlarged. In addition, the relevance and information provided in several network plots (which are generally not readable) is questionable.

A5) *We have extensively revised the figures to improve readability, increase base font size and remove redundancy. The large network plots have been removed to facilitate this.*

R6) Similarly to major Comment 4, findings shown in the figure should be mentioned and explained in the text

A6) *We have ensured all findings are referred to and explained in the text*

R7) Figure 1E: It is mentioned in the text and in Extended Data Figure 2B that 4 cases were sampled twice, but why is this not also visible in Figure 1E?

A7) *We have added the indicators for the second lavage to figure 1E and noted this in the legend.*

R8) Figure 1I: CXCL8 and Il-1b increase in Pn3 should be mentioned in the text.

A8) *We have added a line to the section on Pn3, lines251-253 now read* "Both IL-1β and CXCL8, which play important roles in recruiting neutrophils to the alveolar space, were elevated at transcript and protein level (Figure 2B and 4E)"

R9) Figure captions (e.g. TF enrichment (up/down) in Figure 2C/D, 3C/D, 4C/D) would help the reader to understand the paper better.

A9) *All the figure legends have been revised to reflect the revised figures and to improve clarity. We have moved the transcription factor network diagrams to extended figure 5, and grouped them vertically by "up" or "down-regulated" and horizontally by pneumotype.*

R10) Figure 5 & 6C: The X labels/Y labels showing the gene modules e.g. "BMEpurple" are making the figure not very informative and should be renamed to corresponding names of enrichment patterns (e.g. "Cell cycle processes").

A10) *On this point we respectfully disagree with the reviewer. WGCNA is routinely reported by the arbitrary module colour rather than by the top pathway or enrichment pattern (see the original methods paper: https://doi.org/10.1186/1471-2105-9-559, and further examples: https://doi.org/10.47895/amp.vi0.3934, https://doi.org/10.3389/fimmu.2023.1184700, https://doi.org/10.3389/fimmu.2022.848759). As can be seen in Extended Data Figure 6 each module maps to a range of pathways and enrichment terms, and selecting a single term would give an inaccurate representation of the nature of that module. We would therefore prefer to stick with the standard reporting for WGCNA, retain the module colour names as the reporting approach and then describe the module contents. We do recognise that WGCNA can be somewhat challenging for readers unfamiliar with it to interpret at a glance, however it is particularly useful in this context allowing comparison between 8 subgroups (Pn1-3, B1-2, SRS1-3, with both blood and BAL sequencing in Pn1-3 and B1-2). Head-to-head comparisons for this many groups would be very unwieldly to interpret and present succinctly.*

R11) Figure 6D: Although there are different Pneumonia Types shown in the legend, mentioned in the text and used by the study from Grant et al., the figure only shows COVID-19 Pneumonia types. Is this really correct? If yes, the authors should clarify this in the text and comment on how this fits with their study, where e.g. Pn3 clearly mainly bacterial pneumonia cases.

A11) *We apologise for the confusion caused by our initial version of this plot. The boxplots and PCA plot now use the same symbols for the various types of patient sampled, ensuring consistency between them and showing more clearly how the phenotypes in the Grant cohort distribute across the PCA plot, how they differ by causative pathogen and how they differ in terms of macrophage gene expression profile.*

R12) Extended Data Figure 3 – Could the numbers also be normalized to the volume of BALF? In this regard, it might give additional information to show percentages of cells.

A12) Results of the raw vs normalized analysis shown below. The significant results (macrophages, neutrophils and red cells) are unchanged and of similar magnitude. We are

happy to include either as the final figure, at present we have left the original figure (with hypothesis testing results as noted in A3 above.

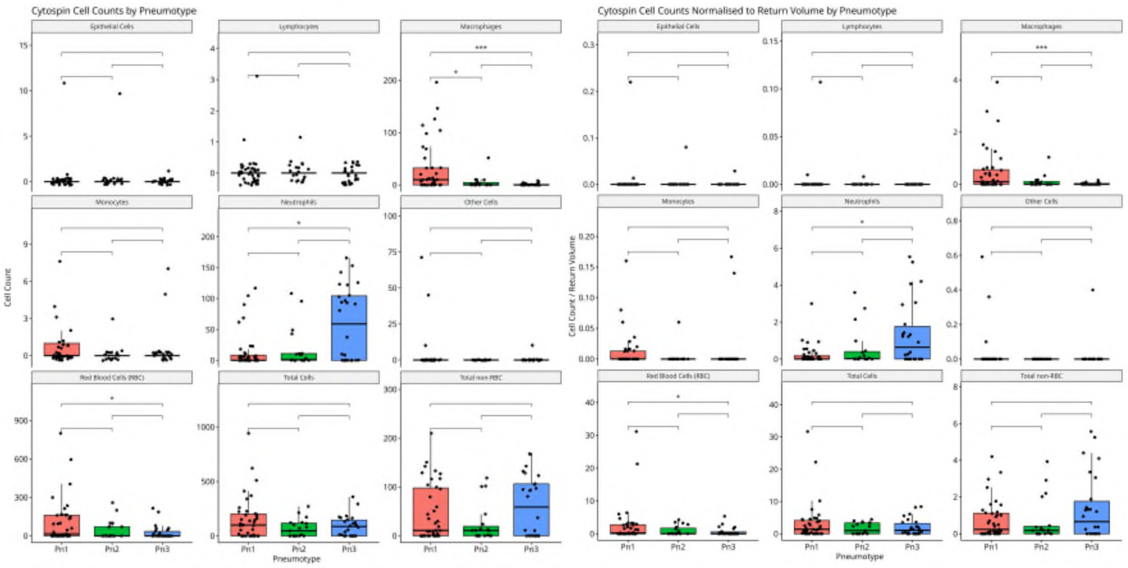

R13) According to the methods, the authors sequenced the BALF cells after spinning them down, but in the text it is written multiple times "sequencable lavage". For clarification, this wording should be rephrased to "sequencable lavage cells" or "BALF cells".

A14) *We have corrected this*

R15) Since plasma cytokines (which were sampled in this study, see Methods Line 897) are not the same as serum cytokines, it should be written plasma cytokines throughout in the study.

A15) *We have corrected this*

Reviewer 3
R16) Figure 1A. A clearer description of elements used to separate each Pn subtype should be provided to clarify the rationale for the distinct phenotypes because all other comparisons rely on the original observation.

A16) *We have revised the figure legend for Figure 1A to read "**A-D** Principal components 1 and 2 of the 10% most highly variable genes from bronchoalveolar lavage plotted with individuals coloured by:* **A** *Pneumotype identified through hybrid hierarchical k-means clustering from BAL bulk RNA transcriptome" to make the rationale clear.*

A17) 2) Figure 1G. Within the first one year post-extubation, nearly ¼ of all Pn types die, and one-year survival ranges from 50 to 75%. This poor overall outcome is not consistent with the rate of survival in the 40-70 year old age groups. Are there any specific factors in this cohort that separate them from other studies?

A17) *The overall mortality rate in our study was 33% (range 26-41% across the Pns). This is consistent with the literature reported mortality rates for severe pneumonia requiring ICU admission and mechanical ventilation (for examples see Cillóniz C, Torres A, Niederman MS.*

Management of pneumonia in critically ill patients. BMJ. 2021 Dec 6;375:e065871. doi: 10.1136/bmj-2021-065871. Papazian L, Klompas M, Luyt CE. Ventilator-associated pneumonia in adults: a narrative review. Intensive Care Med. 2020 May;46(5):888-906. doi: 10.1007/s00134-020-05980-0). Although debate continues regarding to what extent the mortality can be attributed to the pneumonia itself, especially in the cases of secondary/hospital-acquired pneumonia, this does not change the overall mortality as reported. We have added a line to clarify this and added the two references Line 158-159 now reads" In-hospital mortality was 33% which is typical of pneumonia requiring ICU admission and mechanical ventilation [19,20]."

R18) 3) Figure 1I. It is unclear why the authors chose to compare some of the groups. For example, BAL and serum concentrations are randomly compared among Pn1, Pn2, and Pn 3 groups.

A18) We agree with the reviewer that figure clarity can be improved (see A20 below as well). All comparisons were between BAL cytokines alone or plasma cytokines alone. In order to improve this we have separated BAL and plasma cytokines into two rows in revised figure 2B and adjusted P values have been indicated for all comparisons within a given sample type. The selection of cytokines displayed was intended to cover the ones with most relevance to the pneumotypes, we had included all cytokine data in the data supplement and have now placed these into tables (Extended data table 1 and 2, showing BAL and plasma respectively).

R19) Figure 2A. Their analysis based on data shown in Fig 1 points to Pn2 as more distinct than Pn1 and Pn3. Therefore, the rationale for comparing the Pn3 group to the combined two groups is unclear.

A19) *We opted for a 1vs Other over a 1vs1 approach (comparing each Pneumotype to "not that pneumotype", so Pn1 is compared to Pn2/3, Pn2 to Pn1/3, Pn3 to Pn1/2) for differential gene expression. Early during the analysis we did apply both 1vs1 and 1vs Other but found the subsequent pathway mapping was largely synonymous, and that transcriptional differences on a 1vs1 basis were also described in the WGCNA analysis (Extended Data Figure 6 for BAL and Figure 5 for plasma). We therefore felt presenting 3 differential expression comparisons, 1 for each pneumotype was clearer. The authors would not want to suggest that one group is "more" distinct than the others at this stage, as each has distinct clinical and immunological characteristics, and therefore presenting a consistent analysis of each pneumotype was our priority.*

R20) Figure 2E. The large network of interactome could be reduced to increase clarity.

A20) *We have removed the network plots from the figures for clarity.*

R21) 6) Extended data Figure 2B. Three out of 4 repeat BAL sample analysis shows they changed the Pn category from Pn3 to Pn 2 or Pn1. Can the authors speculate how this dynamic change in Pn endotype could be interpreted in the overall scheme of their cohort analysis?

A21) This consistent positive movement along PC2 with time is interesting. The magnitude of this movement also appears to be proportional to the sampling of interval in the 1 case with a longer interval. There is an interesting parallel with https://doi.org/10.1126/scitranslmed.aaa5993 *(fig 2) which shows a 3 dimensional migration*

of blood transcriptomic responses septic patients over time. The tendency towards resolution (Pn2) or an immunosuppressed tissue phenotype (Pn1) would fit with the existing conceptualization of competing pro- and anti-inflammatory responses during sepsis leading many patients to a prolonged phase of impaired immunity after the initial insult (https://doi.org/10.3389/fmed.2021.628302 figure 1). However, with the small number of patients involved and the limited number of time-points we want to be cautious of over-interpreting these changes. We plan to conduct serial sampling in future work to explore this finding. The discussion has been expanded to included this (paragraph lines 501-508), with the references included as new references 58 and 59. The paragraph reads "In the 4 patients who were re-sampled, there was a consistent positive migration in Principal Component 2 (Extended Data Figure 3B), away from Pn3 and towards either Pn1 or Pn2. However, we did not see an overall relationship between duration of illness and pneumotype (Extended Data Figure 3A). Predictable transcriptional time related shifts in blood samples during sepsis occur[58] and the observed migration from a neutrophil driven response to either recovery or immunosuppression fits with current models of immunopathology in sepsis[59]. Although this limited number of repeat samples should only be interpreted as hypothesis generating."

.

R22) Figure 3. Here, it is unclear why Pn1 is compared to the combined Pn2 and Pn3 since the latter two subtypes show significantly different outcomes.

A22) *see A19 above*

R23) Figure 3G. This diagram could be reduced to improve clarity.

A23) *Network plots removed (see A20 above)*

R24) Figure 4. This is perhaps the most important analysis because here the authors compare the cohort that has different outcomes from Pn1, and Pn3 with similar clinical outcomes. However, much like the other gene/protein interactome data in Fig 2E and Fig. 3G, the diffuse data presentation is difficult to comprehend.

A24) *As noted in A21 we have now removed the large network plots from the figures, although we used the gene/protein interactome data to help interpret the nature of the pneumotypes identified, the essential information is conveyed in the differential gene expression and our interpretation has been included in the text of the results.*

R25) Figure 5. The main information embedded within this figure is difficult to discern. Do the authors believe that blood-based genomics and proteomics do not distinguish Pn1, Pn2, and Pn3? Is a higher or lower confidence level attributable to BAL vs blood biomarkers?

A25) *The reviewer raises an important point here and we have sought to clarify our interpretation of the blood data. Based on our analysis we did not find a convincing way to identify Pneumotypes from the measure blood proteins or bulk RNAseq. There is a limited transcriptomic blood signature for Pn3 (upregulation of blood Red and Lightgreen modules and downregulation of Turqouise and Grey60). This is most likely a product of the higher proportion of B1/SRS1 systemic responses in this group, but importantly 60% of Pn3 did not*

have this systemic response. This observation forms the basis of our argument for the "Compartmentalisation of lung responses" [line 346-389]. the description for Figure 5 has been revised to clarify and highlight this conclusion and include a more in-depth description of the figure.

To address the specific comments:

Reviewer 2

R1) While several of our concerns have been satisfactorily addressed by the authors and the overall readability of the paper has improved, one major concern remains. The validation of Pneumotypes in an independent dataset would considerably strengthen the findings and potentially confirm the importance for future use (See A2). Further, although improved, Figure design and flow of results require serious attention.

A1) We have now undertaken the further validation in the suggested external dataset. We have also revised the figures as indicated below, and revised the results to improve flow.

Concerns:
R2) Validation of Pneumotypes
The authors` efforts to identify suitable RNA-seq datasets are commendable, and it is unfortunate that no appropriate datasets were made available. However, we do not agree with the authors` argument that an additional COVID-19 dataset is useless, because their comparison with the Grant et al. dataset (of COVID-19 patients) display a distinct phenotype. In our opinion, Grant et al. data are RNA-seq of macrophages from COVID-19 patients, which is technically different from bulk RNA-seq from BALF (this study). We therefore repeat our earlier suggestion to validate the data using pseudobulk analysis of the suggested single-cell RNA-seq dataset (Wauters et al.), which includes 22 COVID-19 and 13 non-COVID-19 pneumonia patients (Wauters et al., https://doi.org/10.1038/s41422-020-00455-9). The authors have neither

attempted to analyze nor addressed this suggestion in the point-by-point response, and we believe it could substantially strengthen this study.

A2) We thank the reviewer for their comment, and agree that the original validation datasets (Grant and Langellier) were derived from sample types that were quite different and therefore do not fully replicate our original data. We reviewed the Wauters dataset again and there were a number of differences between this and our own, namely the dominance of COVID-19 as an underlying condition, the variable severity (40% were not mechanically ventilated at time of bronchoalveolar lavage), the volume of lavage used (20ml) and the use of single cell RNA sequencing and incomplete overlap with detected genes (28.5% of genes detected after filtering in our BAL samples were not detected in the Wauters data, although 95.8% of genes detected by Wauters were in our BAL samples). However, whilst cognisant of these differences, we have undertaken the suggested analysis and reassuringly have found a number of correspondences with our discovery cohort data that validate our original findings.

We undertook several analyses of the Wauters dataset. First, we pseudobulked the single cell data to approximate our initial bulk RNA data. Applying the same techniques as we did to the discovery data, we identify 3 clusters that we designated W1-3 (shown in revised Figure 6A). Using these clusters we find strong correlation between the gene expression in W2 and Pn2 (Pearson coeff $r=0.742$). Whilst the correlations between W1 and Pn1 and W3 and Pn3 ($r=0.135$ and $r=0.384$ respectively) are weaker, further analyses of these cluster correspondences find relationships to the respective pneumotypes. When we restrict analysis to differentially expressed genes, using the 1-vs-other approach adopted in our discovery cohort, we find strong concordance in direction of expression (W1/Pn1 149/154 shared DEGs, 96.8% concordance , W2/Pn2 2170/2177 shared DEGs, 99.7% concordance, W3/Pn3 2042/2410 shared DEGs 85% concordance). We correlated gene

expression in each W cluster with the modules derived from WGCNA analysis of the discovery cohort (revised Figure 6B). The green, lipid metabolism, module correlated with Pn1 is also correlated with W1. The Blue, cilial assembly, module correlated with Pn2 correlates with W2 whilst the Turquoise and and Magenta modules that anti-correlate with Pn2 show similar anti-correlation with W2. The Turquoise and Magenta modules (innate immunity) correlate with Pn3 and W3. We do, however, find divergent signals between Pn3 and W3 in Cyan, Brown and Salmon (Cell cycle, mitochondrial and translation modules).

We then used the original single-cell RNA major cell type annotations from the Wauters dataset to explore how these related to the cell types identified in the discovery cohort by deconvolution (revised extended data figure 8A). Here, again, there were notable correspondences. W1 had an expansion in alveolar macrophages (similar to the 'M2 macrophages' identified by xCell and the tissue resident macrophage gene signatures upregulated in Pn1). W2 had an expansion in epithelial cells (similar to Pn2 deconvolution), whilst W3 had expansion in neutrophils, inflammatory macrophages and monocytes. Overall this reinforces the hypothesis that the pneumotypes we identified are driven by distinct cell types. Using the clinical meta-data from Wauters supplied under the data-sharing agreement we found further correspondences. W1 was enriched for patients with immunosuppression (46%, vs 25% and 21% in W2 and 3 respectively) whilst bacterial pathogens were enriched in W3 (50%, vs 31% and 25% in W1 and 2 respectively). As with the discovery cohort, neither of these features were exclusive to one cluster or another (clinical metadata is summarised in Extended Data Table 4).

Finally, the single-cell nature of the Wauters dataset allowed us to explore two of the hypotheses we had advanced in the previous version of the manuscript, namely the presence of pro-repair distal alveolar stem cells (DASC) in Pn2, and immature neutrophils (notably IL1R2+ve neutrophils) in Pn3. As Wauters and colleagues did not use these annotations in their

original analysis, we re-labelled the Wauters cells using genes associated with DASCs (*SOX2, SOX9*, *KRT5, TP63,GSTA2, GSTA1, LMO3, PPARGC1A, RPS15A, ALDH1A1, SCGB1A1, TF, GOLGA8A, ATP5MG* -identified from the published DASC literature and cited as revised references 44-46) and the neutrophil maturity signatures identified by Kwok and colleagues (reference 26). Interestingly we found DASCs were enriched in W2, whilst most immature neutrophil subsets including IL1R2+ were enriched in W3 (revised Extended Data Figure 8), consistent with our earlier hypotheses derived from the bulk RNA signatures.

Overall, despite the differences in cohort and technical approaches between Wauters and our discovery cohort, we find recapitulation of multiple features of the pneumotypes in the Wauters data. This combines with our previous validation in the Grant and Langellier data to provide triangulated evidence for these lung-compartmentalised phenotypes with distinct underpinning mechanisms.

We set out the new data from the analysis of the Wauters dataset in Results lines 414-422 and 428-459 alongside revised Figure 6 and Extended Data Table 4 and Extended Data Figure 8. The methods used to describe this approach are set out in lines 1140-1168. We also looked for evidence of *SOX2* and *SOX9* in the discovery cohort pneumotypes, finding both to be upregulated in Pn2 (now noted in results lines 348-350).

R3) Figures and readability

We appreciate that the authors have added statistical tests, the proposed clarifications, and revised most figures to improve readability. These changes are clear improvements. Nevertheless, several figure panels remain difficult to interpret due to their small size and font. It may be helpful to print out the figures to assess which elements are easily readable and which are not. Another approach could be to ask colleagues or non-experts to look at the figures and indicate whether they can

quickly grasp the main message and details. Simplifying or enlarging certain panels and prioritizing selected results to display in the main figures could greatly enhance clarity. Ultimately, figures are most effective when they can be understood at a glance without overwhelming the reader.

In addition: while the results section has improved overall, the narrative still jumps back and forth between figures when the findings are explained. A more linear organization would likely make the text much easier to follow.
Also, the figure legends have been revised and are more informative, but we suggest focusing them on describing the methods and figure-content, while leaving interpretation to the main text.

A3) We thank the reviewer for their positive response to our previous revisions, but acknowledge that there are further changes that can improve clarity whilst not sacrificing too much information.

We have revised Figure 4 and moved the cytokine ratios and peripheral blood neutrophil counts to Extended Data Figure 6. Figure 5 has been reformatted, the title for figure 5A has been changed to "cluster correlation with blood gene modules" for clarity (see R4 below). We have revised figure legends to ensure that interpretation is contained in the main text.

We have revised the panel orders in Figure 4 and Extended Data Figure 5 to ensure they match the text and have ensured that the additional figure (revised Extended Data Figure 8) does the same. We have reviewed the entire text for readability and flow, and made a number of edits to improve these.

R4) WGCNA module analysis
The authors` rationale for using this analysis to compare the different BALF, blood and SRS clusters on a larger scale is understandable. However, Figure 5 only presents blood RNA clusters of the 3 Pneumotypes (while the BALF RNA clusters are compared in Extended Data Figure 6), which should be clearly indicated in the figure description (it is now written “lung and blood cluster correlations” - which is confusing).

A4) We agree that this title was confusing and have now revised it as suggested -see A3 above.

R5) We find the normalized version of the BALF cell counts to be more informative and precise and would recommend replacing the original version. Additionally, it would be more meaningful to label the actual cell concentration (e.g. Cell Count/mL) instead of Cell Count/ Return volume.

A5) We agree with the reviewer and have revised the figure (Extended Data Figure 4) accordingly.

Reviewer #3 (Remarks to the Author):

The manuscript has improved in data presentation and concept. I have no further concerns.

Reviewer #4 (Remarks to the Author):

I co-reviewed this manuscript with one of the reviewers who provided the listed reports. This is part of the Nature Communications initiative to

facilitate training in peer review and to provide appropriate recognition for Early Career Researchers who co-review manuscripts.
